# Bioactive fraction isolated from *Curcuma angustifolia* rhizome exerts anti-diabetic effects *in vitro, in silico* and *in vivo* by regulating AMPK/PKA signaling pathway

**DOI:** 10.3389/fphar.2025.1570533

**Published:** 2025-05-14

**Authors:** P. Kavya, M. Gayathri

**Affiliations:** Department of Bio Medical Sciences, School of Bio Sciences and Technology, Vellore Institute of Technology, Vellore, Tamil Nadu, India

**Keywords:** *Curcuma angustifolia*, bioactive fraction, type 2 diabetes mellitus, molecular docking, AMPK, PKA

## Abstract

*Curcuma angustifolia* Roxb. is a therapeutic herb and a member of the Zingiberaceae family. A potential bioactive fraction was isolated from the methanolic extract of *Curcuma angustifolia* rhizome using column chromatography, and it was characterised using ^1^H-NMR, GCMS and FTIR analyses. The bioactive fraction showed no toxic effects on the HepG2 cell line and it demonstrated inhibition of α-amylase and α-glucosidase enzymes *in vitro with* IC_50_ values of 2.75 ± 0.09 and 4.9 ± 0.07 µM, respectively. Molecular docking analysis also showed that nerolidol, the major constituent of the bioacive fraction inhibits α-amylase and α-glucosidase enzymes competitively, supporting *in vitro* antihyperglycemic activity. ADMET analysis showed that nerolidol has the necessary physicochemical parameters for drug-likeness. It also complies with Lipinski’s rule, indicating that its chemical structure is appropriate for designing safe and bioavailable oral drug. The antidiabetic efficacy of the isolated bioactive fraction was validated in type 2 diabetic albino wistar rats induced with a high-fat diet and a low dose (35 mg/kg bw) of streptozotocin. After 28 days of intervention, the lower and higher doses of the bioactive fraction (100 and 200 mg/kg BW) substantially decreased fasting blood glucose levels and ameliorated hyperglycemia, glucose intolerance, insulin resistance, and hyperlipidemia. The higher dose of bioactive fraction significantly ameliorated liver, kidney, and lipid profiles compared to the standard drug metformin and exhibited lower toxicity in the liver, kidney, pancreas, and epididymal adipose tissue than the lower dose of the bioactive fraction. Gene expression studies revealed that the bioactive fraction upregulated AMPK through downregulating PKA, a mechanism similar to the action of metformin. The results indicate that the isolated bioactive fraction could be a natural alternative to synthetic antidiabetic medications.

## Graphical Abstract

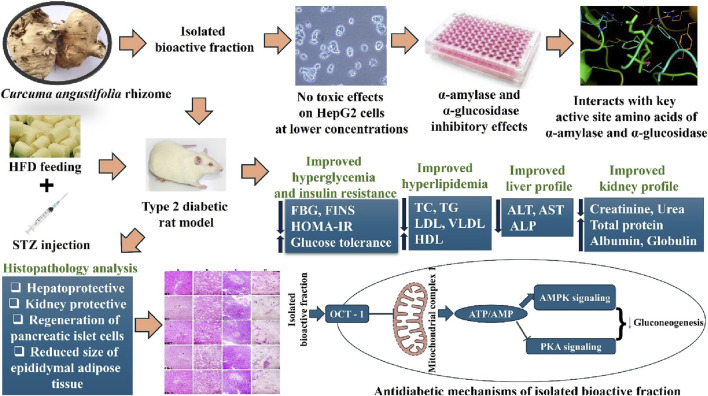

## 1 Introduction

Diabetes mellitus is a complex metabolic condition that impacts around 500 million people globally and is projected to rise to 700 million by 2045 (Harding et al., 2024; Ilic and Ilic, 2024). Diabetes mellitus is defined by either inadequate secretion of insulin or resistance to insulin (Sunil and Mala, 2022). Lack of insulin secretion or inadequate insulin secretion causes type 1 diabetes mellitus, constituting 5%–10% of world diabetes instances (Hafeez et al., 2024). Type 2 diabetes mellitus (T2DM) develops because of resistance to insulin, which is prevalent because of the consumption of high-calorie food and lack of physical exercise (Hassan et al., 2023). T2DM is linked to different diseases, including nephropathy, neuropathy, retinopathy, fatty liver disease, hyperlipidemia, cardiovascular disease, and even alzheimer’s (Kavya and Gayathri, 2024; Petrović et al., 2024).

Adenosine monophosphate-activated protein kinase (AMPK), often called the cellular energy sensor, is a vital therapeutic target for metabolic diseases (Kaewin et al., 2023; Xie et al., 2023). AMPK inhibition leads to the accumulation of lipids in liver cells and reduces mitochondria’s capacity to oxidise free fatty acids (Wang et al., 2021). AMPK activity is decreased during many pathological conditions, including resistance to insulin, hyperglycemia, obesity, Alzheimer’s, and cancer. AMPK activation orchestrates the cellular reaction to energy stress, bringing about favourable physiological changes (Guo et al., 2023). Thus, AMPK regulation may enhance metabolism by reducing the synthesis of triglycerides, free fatty acids, and cholesterol, typically viewed as secondary indicators in managing diabetes mellitus (Wang et al., 2023). AMPK is crucial to enhancing insulin sensitivity, as its deactivation leads to an increase in insulin resistance (Ranganathan and Mahalingam, 2020). Hepatic gluconeogenesis can regulate blood glucose levels (Dai et al., 2024). Consumed glucose is taken into cells by glucose transporters and then converted into glucose-6-phosphate through glucose phosphorylation. This glucose-6-phosphate can then be used in various biochemical pathways (Sim et al., 2020). AMPK directs the glycogen synthesis and glycolysis pathways amid the metabolic pathways (Agius et al., 2020). AMPK activation is necessary for protein kinase A (PKA) to undergo phosphorylation, which has been shown to control various cell functions, such as metabolism, signal transduction, and gene expression (Gao et al., 2021).

Several transcription factors, including hepatocyte nuclear factor 4 and CREB-regulated transcription coactivator 2, play a role in suppressing AMPK-mediated gluconeogenesis (Lee et al., 2010). AMPK prevents the phosphorylation of glycogen synthase, thereby hindering glycogen synthesis. Glycogen synthase is crucial in the process of glycogen synthesis, which is influenced by the allosteric activator glucose 6 phosphate and the process of dephosphorylation (McCorvie et al., 2022). Furthermore, AMPK hinders the process of glycogenesis by phosphorylating glycogen synthase. Through phosphorylation, AMPK initiates the breakdown of glycogen and activates glycogen phosphorylase (Janzen et al., 2018).

Glucagon is secreted by pancreatic α-cells and a high glucagon level is generally observed in T2DM. It is crucial in activating PKA via cyclic AMP (cAMP), leading to hepatic glucose production through the gluconeogenesis pathway (Ranganathan and Mahalingam, 2020). Glucagon utilises the adenylate cyclase to convert ATP to cAMP, activating PKA. PKA has a critical role in controlling the transcription of genes related to gluconeogenesis, which enhances the production of hepatic glucose by impeding glycolysis (Gao et al., 2021). Inhibition of mitochondrial complex-1 decreases the production of ATP. It elevates the cytoplasm’s ADP level, which activates AMPK (Agius et al., 2020). When AMPK is activated, it prevents adenylate cyclase activity, stopping the impact of cAMP. This results in the halting of gluconeogenesis activated by PKA and other transcription processes regulated by PKA (Jayarajan et al., 2019).

Many synthetic medications are commercially available for managing T2DM, including isophane insulin, metformin, and glibenclamide, which are approved in nearly every nations (Alhamhoom et al., 2023). These medications effectively manage T2DM but often cause adverse side effects such as insulin-related weight gain, glibenclamide-induced hypoglycemia, and metformin-linked gastrointestinal issues (Petrović et al., 2024). Also, these pharmacological treatments do not comprehensively target the primary factors contributing to the advancement of secondary diabetic complications (Sunil and Mala, 2022).

Hence, there is a huge necessity for finding natural alternatives for treating T2DM that are effective with minimised side effects (Hassan et al., 2023). The therapeutic effects of medicinal plants are assigned to their secondary metabolites, including terpenoids, polyphenols, flavonoids, alkaloids, glycosides, steroids, tannins, and carotenoids (Akhtar et al., 2023; Suvarna et al., 2023). These compounds have been scientifically proven to have anti-diabetic effects by either increasing insulin secretion from the pancreas or reducing insulin resistance (Akhtar et al., 2023). Compounds purified from medicinal plants have also been found to be more effective against T2DM compared to crude extracts (Kumar et al., 2022).

Metformin, the primary oral medication for T2DM, indirectly activates AMPK and inhibits PKA and its subsequent targets (Ranganathan and Mahalingam, 2020). These studies indicate that activating AMPK may represent an effective approach to reinstate normal gluconeogenesis in individuals with T2DM. In our previous study, the methanolic extract of *Curcuma angustifolia* rhizome exhibited antihyperglycemic activity *in vitro* (Kavya and Gayathri, 2024). Therefore, the current study aimed to isolate and characterise the potential bioactive compound from the methanolic extract of *C. angustifolia* rhizome, demonstrate it is *in vitro* and *in silico* antihyperglycemic potential, and explore its molecular mechanism alleviating T2DM via the AMPK/PKA pathway by measuring physiological and metabolic parameters in high-fat diet (HFD) and streptozotocin (STZ) induced albino wistar rats.

## 2 Materials and methods

### 2.1 Materials

Human hepatoma (HepG2) was procured from the National Centre for Cell Science in Pune, India. Streptozotocin (STZ), 3-[4,5-dimethylthiazol-2-yl]-2,5 diphenyl tetrazolium bromide (MTT), and metformin were procured from Sigma Aldrich in Bengaluru, Karnataka, India. Trizol was purchased from Takara in Joto-ku, Osaka, Japan. α-amylase, α-glucosidase, potato starch, para-nitrophenyl glucopyranoside, dinitro salicylic acid, formalin, ethanol, complementary DNA (cDNA) synthesis kit, and quantitative real-time polymerase chain reaction (qRT-PCR) mix were purchased from HiMedia, Laboratories Pvt. Ltd., in Mumbai, Maharashtra, India. Triglycerides (TG), total cholesterol (TC), low-density lipoprotein cholesterol (LDL-c), high-density lipoprotein cholesterol (HDL-c), alanine transaminase (ALT), alkaline phosphatase (ALP), aspartate transaminase (AST), insulin, urea, creatinine, total protein, and albumin assay kits were purchased from span diagnostics in Surat, Gujarat, India.

### 2.2 Plant collection, authentication, and extraction

Rhizomes of *C. angustifolia* were collected from October to January in the years 2021, 2022, and 2023 from Kasargod, Kerala and authenticated with voucher number C161122049A by experts at the Siddha Medicinal Plants Garden, Ministry of AYUSH, Mettur, Tamil Nadu. The collected rhizomes were dried in the shade and ground into powder using a mixer grinder. Methanolic extract of *C. angustifolia* rhizome was prepared by mixing 10 g of *C. angustifolia* rhizome with 100 mL of methanol (1:10) using a soxhlet apparatus and concentrated with a rotary evaporator at 45°C (Cole-Parmer RV-200-2-120) (Kavya et al., 2024).

### 2.3 Extraction and isolation of bioactive compounds

The concentrated methanolic extract of *C*. *angustifolia* rhizome was mixed with distilled water and filtered through the Whatmann filter paper. The filtrate was combined with an equal amount of n-hexane and stirred for 6 h at 60°C. The upper layer of the mixture (hexane extract) was then separated. 5 g of the concentrated hexane extract was introduced into a glass column containing a silica gel slurry (60–120 mesh), employing a solvent system of n-hexane and ethyl acetate (9:1) as the mobile phase. Fractions ranging from 1 to 53 were collected, and the minor fractions were analysed using thin-layer chromatography (TLC) on a 20 × 20 cm plate. The fractions obtained through TLC were introduced into the column, and column chromatography was repeated using elution solvents of hexane and ethyl acetate (9:1 vol/vol). The obtained fractions were reanalysed using TLC, and the TLC plates were observed under UV light at a short wavelength of 254 nm. The subfractions collected were further purified by repeatedly passing through a silica gel column using a mixture of hexane and ethyl acetate (9:1 vol/vol). The fractions that showed comparable retention factor (R_f_) values were combined to make major fractions (Gonfa et al., 2020; Ugheighele et al., 2020). *In vitro* antihyperglycemic effects were tested for screening of the isolated fractions. Then, the isolated fraction with comparatively higher antihyperglycemic effects was subjected to proton nuclear magnetic resonance (^1^HNMR), gas chromatography-mass spectrometry (GC-MS), and fourier transform infrared spectroscopy (FTIR) analyses for its structure elucidation.

### 2.4 Cytotoxicity assay

The isolated bioactive fraction was introduced to HepG2 cells at varying concentrations (2, 4, 8, 16, 24, 32 µM), and the cells were then maintained in exposure to it for 24 h at 37°C with 5% CO_2_ on a 96-well plate. Then, each well was added with 50 µL of MTT with phosphate-buffered saline (PBS). After mixing, the mixtures were kept at 37°C with 5% CO_2_ for 3 h. After the supernatant was discarded, 100 µL of dimethyl sulfoxide was mixed. The produced formazan was then dissolved by gently shaking the plates. The absorbance was read utilising a microtiter plate reader at 540 nm. The test was run three times for every concentration, and the results were then compared to the control, which contained the untreated cell lines (Pasri et al., 2023).

### 2.5 Assessment of antihyperglycemic effects

#### 2.5.1 α-amylase assay

The bioactive fractions and the standard drug, acarbose (positive control), were prepared in 4 concentrations (2, 4, 6, and 8 µM). 0.5 mg/mL concentration of the α-amylase enzyme was dissolved in 0.2 M sodium phosphate buffer at P^H^ 6.9. 1 mL of the sample and standard was added with 1 mL of the α-amylase enzyme and incubated for 10 min at 25°C. The negative control contained 1 mL of 0.2 M sodium phosphate buffer (P^H^ 6.9) instead of sample. The solution was mixed with 0.5 mL of the substrate (1% starch) and kept at 25°C for 10 min. It was then mixed with 1 mL of the 3,5-dinitrosalicylic acid solution and kept in a water bath at 100°C for 5 min to stop the process. The mixture was made up to 10 mL using sodium phosphate buffer, and the absorbance was recorded at 540 nm. The test was run three times for every concentration (Roy and Mahalingam, 2017). The subsequent formula was utilised to determine the α-amylase activity inhibition percentage:

$$\%\text{ of inhibition of }\alpha -\text{amylase}=\frac{\text{Absorbance}\;\text{of}\;\text{control}-\text{Absorbance}\;\text{of}\;\text{sample}}{\text{Absorbance}\;\text{of}\;\text{control}}\times 100$$

#### 2.5.2 α-glucosidase assay

0.075 U/mL of the α-glucosidase enzyme was added with 1 mL of the sample and standard, acarbose, (positive control) in 4 concentrations (2, 4, 6, and 8 µM). The negative control contained 1 mL of 0.2 M sodium phosphate buffer (P^H^ 6.9) instead of the sample. 0.5 mL of p-nitrophenyl glucopyranoside (3 mM) was mixed to start the process and kept at 37°C for 25 min. Then, 1 mL of Na_2_CO_3_ (0.02 M) was mixed and kept at room temperature for 10 min to halt the process. The mixture was prepared to a volume of 10 mL, and the absorbance was recorded at 400 nm. The test was run thrice for every concentration (Roy and Mahalingam, 2017). The equation used to calculate the inhibition percentage of α-glucosidase activity is as follows:

$$\%\text{ of inhibition of }\alpha -\text{glucosidase}=\frac{\text{Absorbance}\;\text{of}\;\text{control}-\text{Absorbance}\;\text{of}\;\text{sample}}{\text{Absorbance}\;\text{of}\;\text{control}}\times 100$$

### 2.6 Molecular docking analysis

The isolated compound was docked with α-amylase and α-glucosidase receptors, which are involved in the development of hyperglycemia. The protein data bank files of receptors were obtained from the research collaboratory for the structural bioinformatics protein data bank website. The ligand was downloaded in sdf format from the pubchem database (http://pubchem.ncbi.nlm.nih.gov/). Grid boxes were generated, and docking was performed via autodock vina through the PyRx virtual screening tool. Every ligand’s binding affinity (kcal/mol) was noted, and interactions between proteins and ligands were visualised via PyMol and biovia discovery studio (Quimque et al., 2022).

### 2.7 Absorption, distribution, metabolism, excretion, and toxicity property analysis

Absorption, distribution, metabolism, and excretion (ADME) characteristics of the compound were analysed using the SwissADME program (http://www.swissadme.ch/), and its toxicity was analysed using the ADMET Lab server (https://admet.scbdd.com/) (Kavya et al., 2024).

### 2.8 Animal ethics, maintenance, high-fat diet, and streptozotocin induction

10–12 weeks old albino wistar rats (male and female) weighing 180–200 g were procured from the animal house, Vellore Institute of Technology, Vellore. Rats of both sexes were chosen for the study to understand how biological sex influences diabetes risk and treatment responses. The animals were housed and maintained following the guidelines recommended for this experiment by the Institutional Animal Ethical Committee and Committee for the Purpose of Control and Supervision of Experiments on Animals (CPCSEA) with voucher number (VIT/IAEC/24/July23/04). A cycle of 12 h of darkness and light at 25°C ± 2°C with a relative humidity of 40%–60% was provided to the rats in polypropylene cages. The rats were given access to water and pellet diet. The CPCSEA approved a total of 36 rats for the study (18 males and 18 females). We were also provided with 2 rats extra (1 male and 1 female) from the animal house. Hence, the study involved using a total of 38 rats (19 males and 19 females), all of which, with the exception of the normal control group, were given HFD for 6 weeks. The proportion of total kilocalories in the HFD was 17% for carbohydrates, 28% for proteins, and 55% for fat (Ranganathan and Mahalingam, 2020). Following the 6-week period, rats that had fasted overnight received a low dose of STZ (35 mg/kg bw) through intraperitoneal injection, with the exception of the normal control group, which received the citrate buffer (100 mM, pH 4.5). After 72 h from the STZ injection, the animal’s fasting blood glucose (FBG) levels were measured utilising a glucometer manufactured by Johnson and Johnson, specifically the OneTouch Select Simple Glucometer. Rats exhibiting FBG levels ≥ 300 mg/dL were classified as diabetic and chosen for experimentation.

### 2.9 Grouping of rats and experimental design

The rats were assigned to 5 groups in a random manner. The normal control (NC) group consisted of 6 rats, with 3 males and 3 females housed apart. The groups of rats induced with diabetes consisted of 8 rats, with 4 males and 4 females housed apart. Normal saline was given as a vehicle to NC and diabetic control (DC) rats. The diabetic rats were administered orally with the bioactive fraction dissolved in dimethyl sulfoxide at concentrations of 100 and 200 mg/kg bw and the standard drug metformin at 70 mg/kg bw daily for 28 days utilising oral gavage. The rats’ body weight and FBG levels were recorded every week for 28 days.

Group 1: Normal control
Group 2: Diabetic control
Group 3: Diabetic rats + Bioactive fraction (100 mg/kg bw)
Group 4: Diabetic rats + Bioactive fraction (200 mg/kg bw)
Group 5: Diabetic rats + Metformin (70 mg/kg bw)

### 2.10 Measurement of fasting blood glucose level, body weight, water intake, and food intake

The FBG level of all the experimental rats was checked every week utilising a glucometer. The body weight, water, and food intake were also assessed weekly during the study. Water and food intake were determined by subtracting the water/food remaining in the cage from the original quantity provided to each group (Pandeya et al., 2022).

### 2.11 Oral glucose tolerance test

Rats that had fasted overnight (12 h) and had unrestricted access to water were used for the oral glucose tolerance test (OGTT). The treatment groups received glucose solution (2 g/kg bw) orally. Glucose tolerance levels were assessed at 0, 15, 30, 60, 90, and 120 min utilising a glucometer after administration. The subsequent formula was used to determine the area under the curve (AUC) (Li J. et al., 2024):

$$\text{AUC }\left(\frac{\frac{\text{mg}}{\text{dL}}}{h}\right)=\frac{\left({BG}_{0}+{BG}_{15}\right)x\;15}{60}+\frac{\left({BG}_{15}+{BG}_{30}\right)x\;15}{60}+\frac{\left({BG}_{30}+{BG}_{60}\right)x\;15}{60}+\frac{\left({BG}_{60}+{BG}_{90}\right)x\;15}{60}+\frac{\left({BG}_{90}+{BG}_{120}\right)x\;15}{60}$$

### 2.12 Collection of blood and tissue samples

Following 28 days of intervention, the rats were euthanised, and samples of blood and organs were gathered. The blood sample was obtained through the cardiac puncture method. After the blood samples were separated into serum and plasma, after centrifuging for 10 min at 4,000 rpm, they were stored at −80°C until analysis. The liver, kidney, pancreas, and epididymal adipose tissue were gathered from every group, rinsed with 1 × PBS, and preserved in 10% formalin (pH 7). The tissue samples were kept at 25°C up to histopathological analysis. Liver samples were gathered from every group, rinsed with 1 × PBS, and stored in RNA later to isolate RNA.

### 2.13 Assessment of biochemical parameters

Serum biochemical parameters, including fasting insulin (FINS) level, lipid profiles (TC, TG, HDL), liver function markers (AST, ALP, ALT), and kidney profiles (urea, creatinine, total protein, albumin, globulin) were assessed, utilising the commercially available kits in triplicates. Additionally, insulin sensitivity resistance index (HOMA-IR), LDL, very low-density lipoprotein (VLDL), and AI (atherogenic index) were determined utilising the following equations (Ranganathan and Mahalingam, 2020; Pandeya et al., 2022):

$$\text{HOMA}-\text{IR}=\frac{\text{FBG}\;\left(\text{mg}/\text{dL}\right)\;x\;\text{FINS}\;\left(\text{mU}/L\right)}{405}$$

$$\text{VLDL}=\frac{\text{TG}}{5}$$

$$\text{LDL}=\text{TC}-\left(\text{HDL}+\text{VLDL}\right)$$

$$\text{AI}=\frac{\text{TC}-\text{HDL}}{\text{HDL}}$$

### 2.14 Measurement of tissue weight and histopathological analysis

The organs and tissues collected from all the groups of animals, including the liver, kidney, pancreas, and epididymal adipose tissue, were washed in 1 × PBS and weighed. The organs and tissues gathered were stored in 10% formalin (pH 7). After fixation in formalin, the samples were immersed in paraffin and broken into 4 mm sections utilising a rotary microtome. Deparaffinisation of the samples was done utilising xylene, followed by dehydration utilising varying concentrations of ethanol. The tissue samples were treated with haematoxylin and eosin (H&E) stain and inspected using a microscope to observe any histopathological changes, which were then recorded (Li Y. et al., 2024).

### 2.15 qRT-PCR analysis

The messenger RNA (mRNA) from the liver tissue of all experimental groups of rats was extracted using Trizol reagent, and the concentration was measured using nanodrop. The mRNA was then reverse-transcribed into complementary DNA (cDNA) using a Hi-cDNA Synthesis Kit. The reverse transcription reaction involved incubation at 420°C for 60 min, followed by a step at 700°C for 5 min, and then the reaction was terminated by storing the product at 4°C. The cDNA produced was kept at −800°C and promptly utilised for the following qRT-PCR procedure. The qRT-PCR analysis was conducted using the Hi-SYBr master mix (with Taq polymerase) to evaluate the relative gene expression. The qRT-PCR reaction involved an initial denaturation at 950°C for 5 min, 40 cycles of denaturation at 940°C for 10 s, annealing at 55–600°C for 45 s, and extension at 720°C for 30 s. The primer sequences of AMPK, PKA, and β-actin are given in Table 1. The mRNA levels of target genes were normalised by the mRNA levels of β-actin using the 2^−ΔΔCT^ method (Ranganathan and Mahalingam, 2020).

**TABLE 1:** Target genes and primer sequences.

Target gene	Forward primer (5′-3′)	Reverse primer (5′-3′)
AMPK	TTT​GCC​TAG​AAT​CCC​CAC​AG	TAA​GGA​GCC​CAG​AAA​ACA​GC
PKA	AGC​AGG​AGA​GCG​TGA​AAG​AG	AAC​AGC​ACA​CTG​TTC​AAG​AGG​A
βactin	TTT​GCC​TAG​AAT​CCC​CAC​AG	TAA​GGA​GCC​CAG​AAA​ACA​GC

### 2.16 Statistical analysis

All the experiments were performed in triplicate, and the data were displayed as mean ± SD. Data analysis was done on JMP Pro 17 utilising one-way analysis of variance and Student’s t-test. The significance of statistics was assessed for p-values less than 0.05.

## 3 Results and discussion

### 3.1 Separation of bioactive fractions by silica gel column chromatography

The methanolic extract of *C*. *angustifolia* rhizome was fractionated using n-hexane, and the crude n-hexane extract was subjected to silica gel column chromatography. The mobile phase was adjusted based on column chromatography and TLC, and the optimal ratio of n-hexane to ethyl acetate was found to be 9:1. 53 minor fractions were collected in total in the corresponding beakers (10 mL each). Each fraction was analysed using TLC to determine the number of bands in the minor fractions. The fractions with comparable R_f_ values were combined, resulting in 14 major fractions. All the concentrated major fractions were analysed for antihyperglycemic effects (Table 2).

**TABLE 2:** Inhibitory potential of the isolated major fractions on α-amylase and α-glucosidase.

Major fraction	Inhibitory effects (%)	
	α-amylase	α-glucosidase
1	0	0
2	13.32 ± 0.87	10.54 ± 1.25
3	11.76 ± 1.78	16.38 ± 2.56
4	20.64 ± 2.41	19.52 ± 1.67
5	11.35 ± 2.32	18.81 ± 2.06
6	17.64 ± 1.84	12.84 ± 1.64
7	0	0
8	69.38 ± 1.44^b^	62.45 ± 1.54^b^
9	22.57 ± 0.99	19.28 ± 2.52
10	20.74 ± 1.26	18.72 ± 1.86
11	31.58 ± 1.85^c^	38.73 ± 2.43^c^
12	0	0
13	2.36 ± 1.69	1.84 ± 2.05
14	3.46 ± 0.96	2.58 ± 1.49

Note: The values are shown as the standard deviation of the mean (mean ± SD), n = 3, p < 0.001.

### 3.2 *In vitro* antihyperglycemic potential of the isolated major fractions

#### 3.2.1 α-amylase and α-glucosidase inhibition effects

**FIGURE 1:**
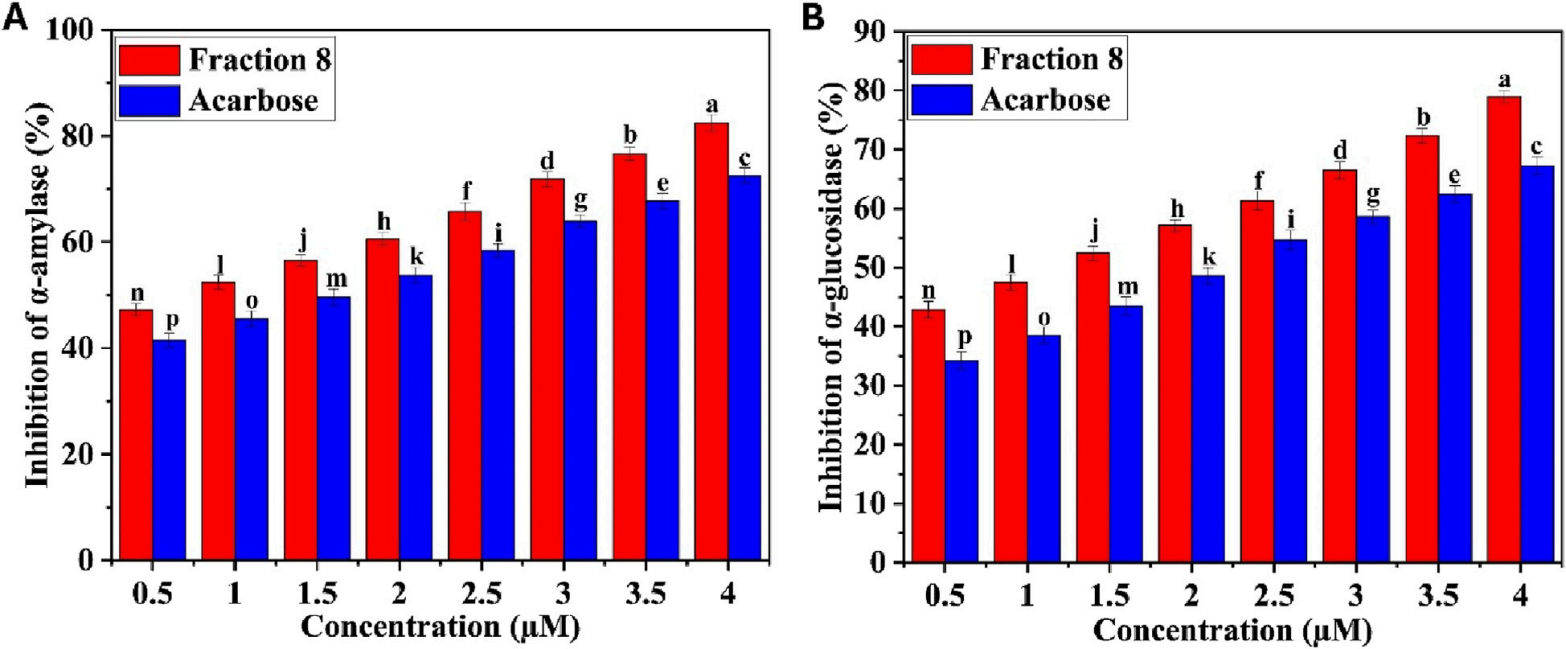
**(A)** α-amylase inhibitory effect of bioactive fraction 8, **(B)** α-glucosidase inhibitory effect of bioactive fraction 8. Significant differences between the tested sample and the standard are indicated by different letters (p < 0.001).

A potential therapeutic strategy for treating hyperglycemia is to inhibit the enzymes that hydrolyse carbohydrates, in particular α-amylase and α-glucosidase, as this leads to the hydrolysis of carbohydrates and elevated blood glucose levels (Gök et al., 2020; Sansenya et al., 2020). The potential of all the isolated major fractions to inhibit α-amylase and α-glucosidase was tested. Major fractions 8 and 11 had higher inhibitory effects on α-amylase and α-glucosidase compared to the other fractions (Table 2). Major fractions 8 and 11 inhibited α-amylase enzyme with inhibition percentages of 69.38 ± 1.44^b^ and 31.58 ± 1.85^c^ %, respectively, and α-glucosidase enzyme with inhibition percentages of 62.45 ± 1.54^b^ and 38.73 ± 2.43^c^ %, respectively. The α-amylase and α-glucosidase inhibition percentages of major fraction 8 (69.38 ± 1.44^b^ and 62.45 ± 1.54^b^ %, respectively) and the standard drug, acarbose (72.48 ± 1.55^a^ and 67.26 ± 0.97^a^ % respectively) were comparable. The potential of fraction 8 to inhibit α-amylase was greater than that of α-glucosidase, as shown by the inhibition percentage (Table 2). Figures 1A,B illustrate how fraction 8 inhibited α-amylase and α-glucosidase depending on its concentration. However, the standard drug acarbose had higher inhibition percentages on α-amylase and α-glucosidase than fraction 8, around 0.04 and 0.07 times, respectively. Fraction 8 exhibited a greater inhibitory activity on α-amylase (IC_50_ value: 2.75 ± 0.09 µM) and a comparatively lesser inhibitory activity on α-glucosidase (IC_50_ value: 4.9 ± 0.07 µM). The standard acarbose inhibited α-amylase and α-glucosidase with IC_50_ values of 2.21 ± 0.07 and 3.28 ± 0.12 µM, respectively. Even though the major fraction 11 had satisfactory inhibitory effects on α-amylase (31.58 ± 1.85^c^ %) and α-glucosidase (38.73 ± 2.43^c^ %) enzymes, it was much less compared to the standard drug, acarbose. The findings show that fraction 8 has higher inhibitory effects on α-amylase and α-glucosidase enzymes compared to the standard drug acarbose.

### 3.3 Effect of bioactive fraction 8 on cell viability

**FIGURE 2:**
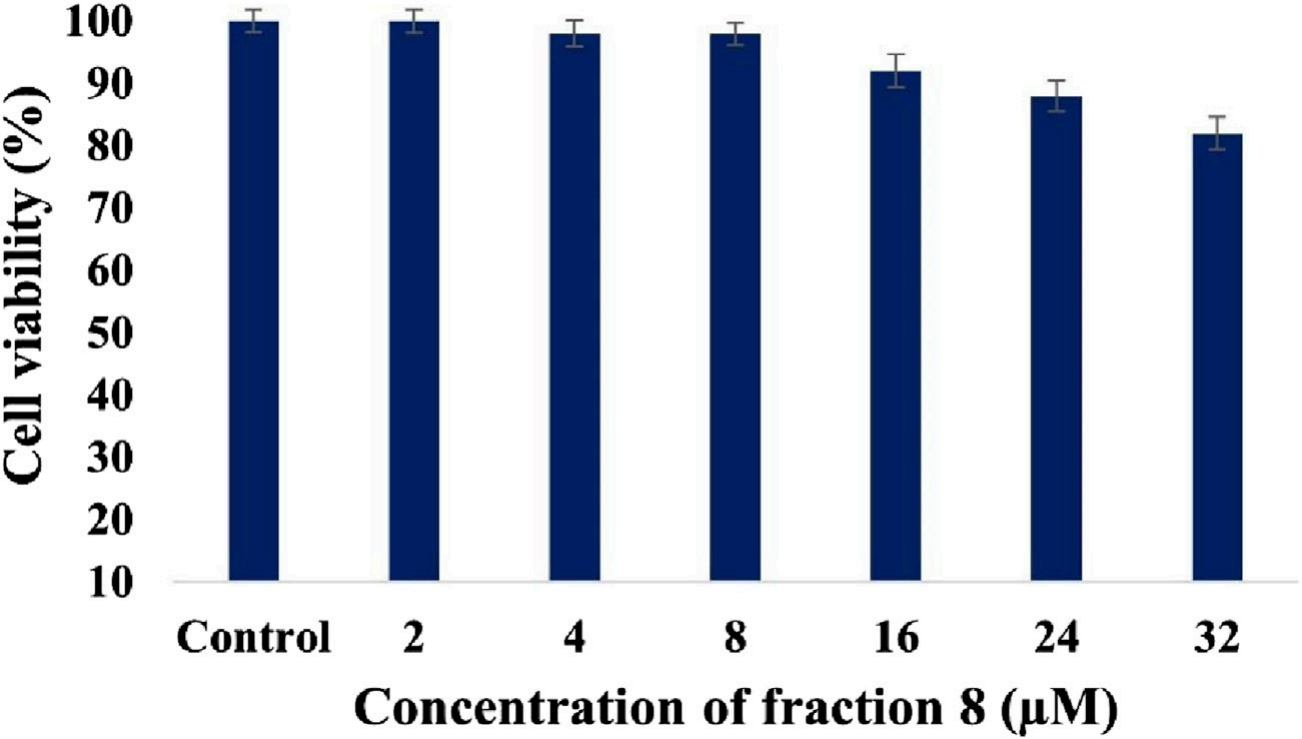
Viability of the HepG2 cell line after treatment with various concentrations of bioactive fraction 8. Significant differences between the tested sample and control are indicated by different letters (p < 0.05).

Cytotoxic effects of bioactive fraction 8 were tested on the human hepatoma (HepG2) cell line employing 3-[4,5-dimethylthiazol-2-yl]-2,5 diphenyl tetrazolium bromide (MTT) assay. Succinate tetrazolium reductase reduces MTT to formazan, which only reacts with live cells and is indicated by purple coloration (Ghasemi et al., 2021; Fajriaty et al., 2024). The results exhibited that bioactive fraction 8 has no toxic effects on the HepG2 cell line. Compared with the control, significant cell viability was monitored for 2, 4, 8, and 16 µM concentrations of bioactive fraction 8, and comparatively decreased cell viability was monitored for 24 and 32 µM concentrations of bioactive fraction 8. Figure 2 shows the viability of HepG2 cells (%) at different concentrations of bioactive fraction 8 (2–32 µM). Based on the absorbance value, the cell viability was 82% for 32 µM concentration of bioactive fraction 8. The findings of the MTT assay indicate that bioactive fraction 8 is nontoxic in HepG2 cell lines, which could be studied to manage T2DM.

### 3.4 Structural elucidation of isolated bioactive fraction 8

**FIGURE 3:**
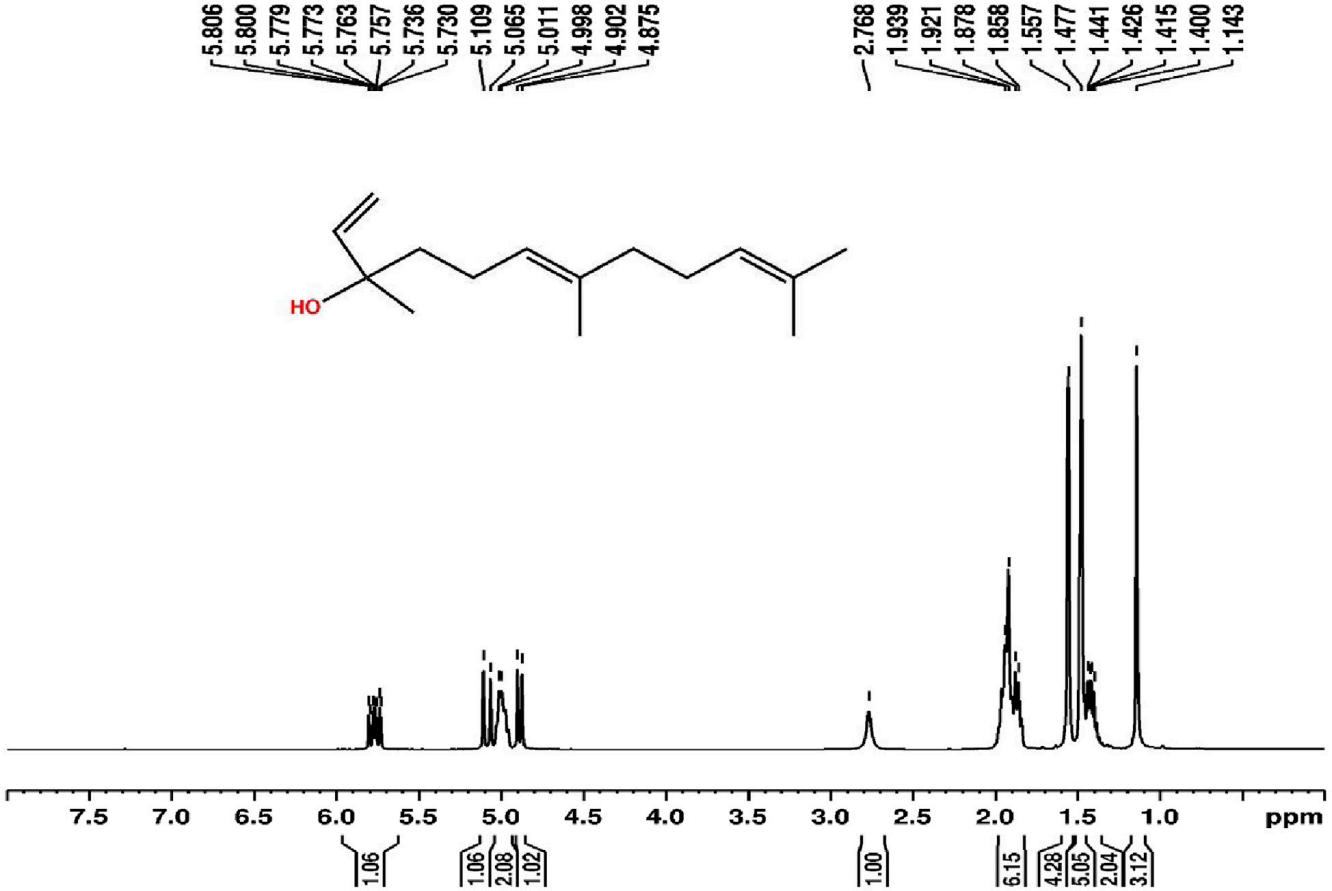
^1^H NMR spectra of bioactive fraction 8.

**FIGURE 4:**
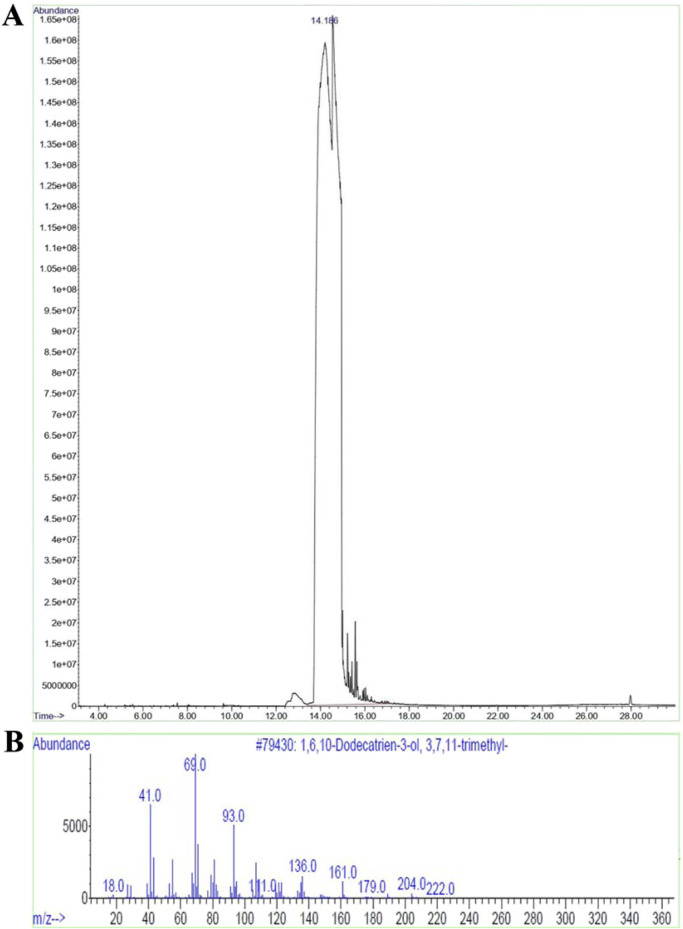
**(A)** GC-MS chromatogram of bioactive fraction 8, **(B)** Mass spectra of nerolidol.

Since the isolated bioactive fraction 8 showed comparatively higher α-amylase and α-glucosidase inhibitory effects and lower toxicity, it was subjected to characterisation using ^1^H NMR, GC-MS, and FTIR analyses. Bioactive fraction 8 was obtained as a pale-yellow colored liquid, approximately 16 mg. The bioactive fraction 8 was identified as nerolidol, with the molecular formula C_15_H_26_O, by ^1^H NMR (Figure 3), GC-MS (Figure 4), and FTIR (shown in Supplementary Material as Supplementary Figure S1) analyses. ^1^H NMR (CDCl_3_, 400 MHz, δ, ppm) 5.806–5.800 (1H, d, J = 1.2 Hz), 5.779–5.773 (1H, d, J = 1.2 Hz), 5.763–5.757 (1H, d, J = 1.2 Hz), 5.736–5.730 (1H, d, J = 1.2 Hz), 5.109–5.065 (1H, d, J = 8.8 Hz), 5.011–4.998 (2H, d, J = 2.6 Hz), 4.902–4.875 (1H, d, J = 5.4 Hz), 2.768 (1H, s), 1.939–1.921 (3H, d, J = 3.6 Hz), 1.878–1.858 (3H, d, J = 4 Hz), 1.557 (4H, s), 1.477 (5H, s), 1.441–1.426 (1H, d, J = 3 Hz), 1.415–1.400 (1H, d, J = 3 Hz). GC-MS: Major peak at the retention time of 14.185 min with peak area percentage of 98.53 and m/z ratio of 222.0. The peak area percentage of 98.53 indicates that the isolated compound, nerolidol, is 98.53% pure. Six other peaks were also present in the GC-MS chromatogram, identified as 3-ethyl-1,5-octadiene, α-caryophyllene, α-farnesene, β-bisabolene, β-caryophyllene and α-tocopherol, with peak area percentages of 1.07, 0.05, 0.07, 0.07, 0.02, and 0.19, respectively. FTIR: 3416.28: O-H (Alcohol), 2966.947: H-C-H (Alkane), 2923.556: H-C-H (Alkane); 2856.06: C-H (Alkane); 1449.243: C-H (Alkyl); 1374.997: CH3 C-H (Alkyl); 1107.904: C-O (Alcohol); 994.1248: C-H (Alkene); 835.9905: =C-H (Alkene); 741.4955: =C-H (Alkene); 688.4627: =C-H (Alkene).

### 3.5 Molecular docking analysis of nerolidol

The interactions of nerolidol with α-amylase and α-glucosidase enzymes were analysed by molecular docking. The analysis results indicate any possible affinities between the compound and the enzymes. The binding energies of nerolidol with α-amylase and α-glucosidase are shown in Table 3, and interactions between the docked nerolidol and the receptors are shown in Supplementary Material as Supplementary Figure S2. Molecular docking results showed that nerolidol exhibits a binding energy of −6.1 kcal/mol with α-amylase. Nerolidol forms conventional hydrogen bonds with ASP197 and GLU233, two critical amino acids for enzyme activity. The standard acarbose showed a hydrogen bond with just one of the crucial amino acids of the α-amylase enzyme, ASP197, exhibiting a binding energy of −6.9 kcal/mol. Acarbose also showed a hydrogen bond with TRP59. Molecular docking with α-glucosidase showed that nerolidol had a greater binding energy of −7.1 kcal/mol to the receptor. Nevertheless, nerolidol showed no hydrogen bonds with the enzyme’s active site amino acid residues. Nerolidol formed a pi-sigma bond with the active site amino acid, PHE166, and with TYR65. Despite having a relatively low binding affinity of 5.8 kcal/mol, acarbose formed hydrogen bonds with two specific active site amino acids, ASP333 and GLU271, and also with LEU227, ASP301, and VAL335. Inhibiting the α-amylase and α-glucosidase enzymes, which convert carbohydrates into glucose, can manage hyperglycemia (Kato-Schwartz et al., 2020). Since nerolidol formed hydrogen bond interactions with the key active site amino acids of α-amylase, it showed a higher binding affinity with the enzyme, and thus, it shows competitive inhibition. In addition, it binds in the same binding site as acarbose. Nerolidol also binds with α-glucosidase with a relatively higher binding affinity and forms a pi-sigma bond with an active site amino acid, and thus, it shows competitive inhibition. Therefore, it is concluded that nerolidol functions as a potential competitive inhibitor of α-amylase and α-glucosidase, aiding in managing hyperglycemia.

**TABLE 3:** Binding affinity of docked nerolidol with α-amylase and α-glucosidase.

Ligands	Binding energy (kcal/mole)	
	α-amylase	α-glucosidase
Nerolidol	−6.1	−7.1
Acarbose	−6.9	−5.8

### 3.6 ADMET analysis of nerolidol

Nerolidol was assessed for ADMET properties (Tables 4, 5). The ADMET analysis uses a series of guidelines derived from knowledge to assess the bioavailability, absorption in the gastrointestinal tract, blood-brain barrier permeability, solubility, inhibition of cytochrome P450, molecular weight, topological polar surface area, molar refractivity, hydrogen bond donors and acceptors, toxicity of compounds. Topological polar surface area affects how molecules move through biological membranes. Compounds with a topological polar surface area of less than 140 Å^2^ can readily pass through membranes, causing fewer side effects (Daoui et al., 2022). Nerolidol has a topological polar surface area of 20.23 Å^2^ and exhibits greater permeability and bioavailability, resulting in lesser adverse effects. Conversely, the standard drug, acarbose, with a topological polar surface area of 321.17 Å^2^, shows lower permeability across the cell membrane and causes more adverse effects. The log P value measures how hydrophobic or lipophilic a compound is. If a compound’s log P value is more than 5, it is considered exceedingly lipophilic. This causes increased toxicity as the drug lasts in the phospholipid bilayer for a longer duration and disperses all over the body. Contrarily, a negative log P value suggests a hydrophilic compound, causing it challenging to absorb the compound. If a compound’s log P value is 5 or less than 5 and not negative, it suggests good absorption and permeation qualities, allowing the compound to move across the hydrophilic layer of the membrane and reach the hydrophobic phospholipid bilayer (Jia et al., 2020; Carrasco-Correa et al., 2021). The Log p-value of nerolidol was less than 5, i.e., in the acceptable range, indicating its bioavailability for oral administration. The log p-value for the standard drug, acarbose, also fell within the normal range. The Log S values of −3.80 and 2.13 suggest that nerolidol and acarbose are soluble in water. The molar refractivity value of nerolidol also falls within the acceptable range of 40–130, whereas acarbose’s molar refractivity value was 136.69. The distribution of drugs all over the body depends significantly on their absorption in the gastrointestinal tract. Once absorbed, drugs are distributed all over the body and must cross the blood-brain barrier to access the central nervous system. Hyperglycemia can influence transport across the blood-brain barrier, leading to insulin, amino acid, choline, and glucose transport changes. Additionally, hyperglycemia can weaken the blood-brain barrier by interrupting the tight connections and inducing oxidative pressure in the small blood vessels of the central nervous system (Banks and Rhea, 2021). Nerolidol has high absorption in the gastrointestinal tract and can cross the blood-brain barrier. In contrast, the standard drug acarbose has low absorption in the gastrointestinal tract and is not able to pass the blood-brain barrier. The swissADME tool was used to analyse the boiled egg graphical plots of nerolidol and acarbose (shown in Supplementary Material as Supplementary Figures S3A, C). In the graphical representation, the egg white portion represents gastrointestinal absorption, the egg yellow portion represents permeation of the blood-brain barrier, and the egg grey region represents alternative routes apart from the oral route. The boiled egg graphs showed that nerolidol has high absorption in the gastrointestinal tract and can cross the blood-brain barrier. Assessing drug-likeness evaluates the bioavailability of a compound as an effective oral drug (Jia et al., 2020). The bioavailability radar of nerolidol demonstrates its drug-like properties (shown in Supplementary Material as Supplementary Figures S3B, D). The pink region of the bioavailability radar shows the necessary range to meet these characteristics. The hydrogen bond donor and acceptor centres help form hydrogen bonds and increase water solubility (Kavya et al., 2024). Nerolidol follows Lipinski’s rule, having a molecular weight of 222.37 g/mol, 4.19 log p, 1 hydrogen bond donor, and 1 hydrogen bond acceptor, displaying drug-like characteristics. Nerolidol follows Lipinski’s rule with no violations. In comparison, acarbose does not comply with Lipinski’s rule and has 3 violations. Acarbose has a molecular weight of 645.60 g/mol, greater than 500 g/mol, suggesting it does not penetrate easily across biological membranes. Acarbose has 14 hydrogen bond donors, greater than 5, and 19 hydrogen bond acceptors, exceeding the normal range 10. CYP enzymes are a group of isozymes responsible for breaking down drugs, steroids, fatty acids, carcinogens, and bile acids (Carrasco-Correa et al., 2021). According to the predicted values, nerolidol does not act as a substrate or inhibitor of CYP enzymes. This indicates that nerolidol will probably break down efficiently in the liver and be removed from the body, reducing the risk of inducing harmful impacts. The findings strongly support the idea that nerolidol could be used as a drug. Additionally, the standard drug acarbose also does not inhibit CYP enzymes. The predicted bioavailability score of nerolidol to drug targets has been determined. A higher bioavailability score shows that the compound is more likely to be active. A bioavailability score above 0.00 shows the compound is anticipated to demonstrate strong biological effects. Scores between −0.50 and 0.00 show moderate activity, while below −0.50 indicate inactivity (Batiha et al., 2020; Carrasco-Correa et al., 2021). Nerolidol has a bioavailability score of 0.55, while acarbose has a lower score of 0.17. Nerolidol is considered physiologically active, possesses high bioavailability, and is more likely to have therapeutic effects. The study of toxicology is essential for understanding how a drug is processed in the body, including its absorption, distribution, metabolism, and excretion (Rim, 2020). Toxicology profiling entails evaluating human liver toxicity, drug-induced liver injury, mutagenicity, and the median lethal dose (LD_50_) for acute toxicity. If a drug induces liver injury during clinical research, the development process might be halted, which can be both unfavorable and costly. Drug-induced liver injury poses a significant risk to patient wellbeing and can result in the withdrawal of drugs from the market (Lin et al., 2022). The predicted probability values for hepatotoxicity show that nerolidol poses a lower risk to the liver. Testing for mutagenicity helps assess whether a substance can cause mutations (Rim, 2020). According to the predicted value, nerolidol is unlikely to be mutagenic or toxic. Evaluating acute toxicity in animals is essential for assessing pharmacovigilance (Kavya et al., 2024). The oral acute toxicity level of nerolidol was at acceptable levels. Acarbose was not found to cause mutations or human hepatotoxicity, but it induces liver damage. Nerolidol has the optimal physicochemical characteristics needed for drug-likeness and lower toxicity. It also complies with Lipinski’s rule, indicating that its chemical structure is appropriate for designing safe and bioavailable oral drug (Banks and Rhea, 2021).

**TABLE 4:** ADME properties of nerolidol.

ADME parameters	Nerolidol	Acarbose
Molecular weight (g/mol)	222.37	645.60
Molar refractivity	74.00	136.69
Hydrogen bond donor	1	14
Hydrogen bond acceptor	1	19
Topological polar surface area (Å^2^)	20.23	321.17
Log Po/w	3.64	1.43
Gastrointestinal absorption	High	Low
Blood-brain barrier permeant	Yes	No
Log S	−3.80	2.13
CYP3A4 inhibitor	No	No
Lipinski’s (violations)	Yes (0)	No (3)
Bioavailability score	0.55	0.17

**TABLE 5:** Toxicity analysis of nerolidol.

Toxicity parameters	Nerolidol	Acarbose
Drug-induced liver injury	0.148	0.678
Ames Mutagenicity	0.05	0.256
Human Hepatotoxicity	0.314	0.418
LD_50_ of acute toxicity	>500 mg/kg	>500 mg/kg

### 3.7 Effect of bioactive fraction 8 on body weight, food intake, and water intake

**FIGURE 5:**
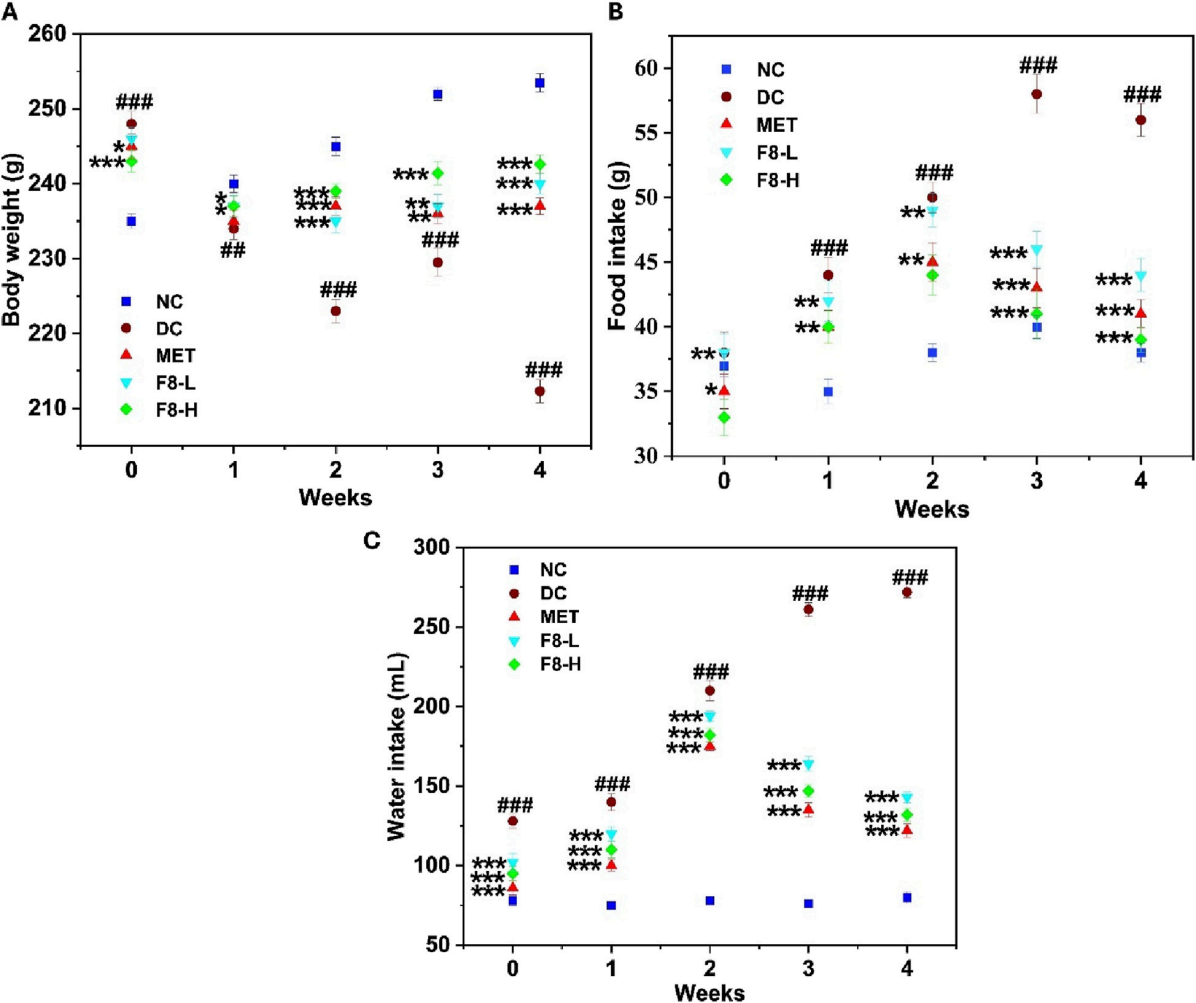
Effect of bioactive fraction 8 on **(A)** body weight, **(B)** food intake, **(C)** water intake. The values are represented as the mean ± standard deviation (n = 6). Results were analysed using one-way ANOVA, and significant differences were denoted as ^###^p < 0.001, ^##^p < 0.01 in comparison with the NC group, whereas ***p < 0.001, **p < 0.01, *p < 0.05 in comparison with the DC group.

There was mortality in groups of rats induced with STZ. After developing diabetes, 2 rats died in each group. In the DC group of rats and the bioactive fraction 8 higher dose (F8-H)-treated group of rats, 1 male and 1 female rat died. In the bioactive fraction 8 lower dose (F8-L) and metformin (MET)-treated groups, 2 female and 2 male rats died, respectively. There were 6 rats in each group after the mortality (3 males and 3 females). The effect of bioactive fraction 8 isolated from *C*. *angustifolia* rhizome on the experimental rats’ body weight, food intake, and water intake was studied. A notable rise in body weight was seen in all the rats before developing diabetes. Upon developing diabetes, the diabetic groups showed a substantial reduction in body weight compared to the NC group (p < 0.001), which is in harmony with the symptoms of diabetes, such as increased thirst, hunger, and inexplicable weight loss. The body weight of the diabetic rats exhibited a gradual increase after being treated with 100 and 200 mg/kg bw of the bioactive fraction 8 from week 2 to week 4, significantly (p < 0.001) compared to the DC group and (p < 0.05) compared to the MET-treated group, as depicted in Figure 5A. (Jiang and Zhang, 2022). also reported that the body weight of the diabetic rats treated with nerolidol increased compared to the diabetic control group of rats. MET-treated groups did not demonstrate substantial control over body weight in comparison with the diabetic rats, and the findings align with prior research (Ranganathan and Mahalingam, 2020). The substantial decrease in weight seen in MET-treated groups may be attributed to impaired fat tissues (Biondo et al., 2018; Ranganathan and Mahalingam, 2020). There were highly significant differences in food and water intake between the NC and diabetic rats (p < 0.001). Following treatment with F8-L, F8-H and MET, food and water intake gradually reduced from week 2 to week 4 (p < 0.001), while rats in the DC group continued to exhibit an increased intake (Figures 5B,C).

### 3.8 Effect of bioactive fraction 8 on FBG level and OGTT

**FIGURE 6:**
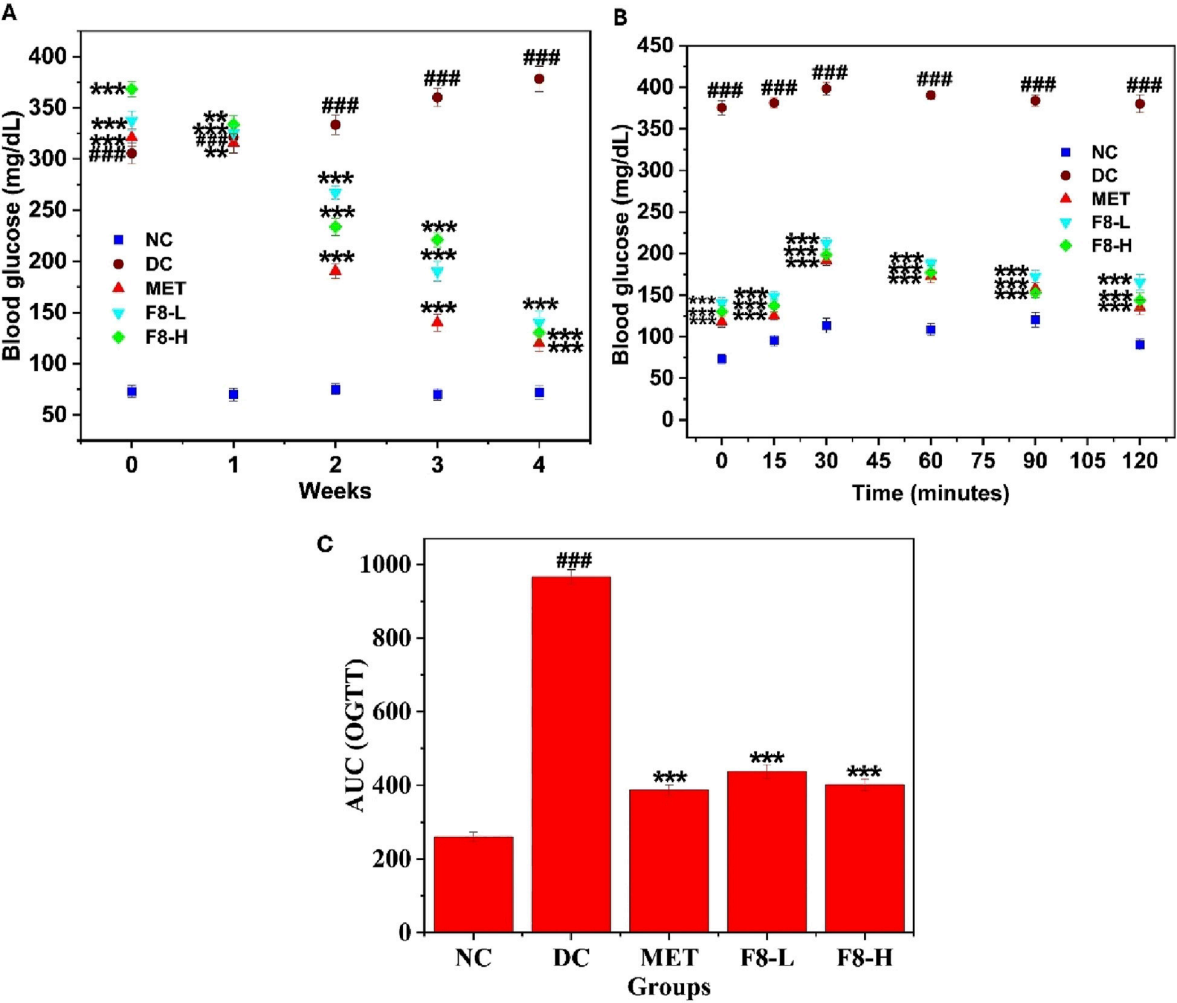
Effect of bioactive fraction 8 on **(A)** FBG level, **(B)** OGTT, **(C)** AUC of OGTT. The values are represented as the mean ± standard deviation (n = 6). Results were analysed using one-way ANOVA, and significant differences were denoted as ^###^p < 0.001 in comparison with the NC group, whereas ***p < 0.001, **p < 0.01 in comparison with the DC group.

The female rats exhibited slightly higher FBG levels than the male rats after 72 h of STZ injection. However, there were no significant differences between the FBG levels of male and female rats. The effect of bioactive fraction 8 on FBG level was assessed for 28 days. Following the induction of diabetes, a notable increase (p < 0.001) in FBG levels was noted in the diabetic groups (305.50 ± 10.29 mg/dL) compared to the NC group (73.12 ± 6.19 mg/dL). When compared to DC, F8-L (140.26 ± 11.19 mg/dL), F8-H (130.43 ± 9.62 mg/dL), and MET (120.17 ± 7.99 mg/dL), treated groups demonstrated a significant reduction (p < 0.001) in FBG levels after the 28 days of treatment. It was previously reported that the blood glucose levels of diabetic rats were significantly reduced after treatment with nerolidol for 4 weeks (Jiang and Zhang, 2022). Both male and female rats showed a reduction in FBG levels after treatment with the isolated bioactive fraction 8 and metformin from week 1 to week 4, and there were no significant differences between the FBG levels of male and female rats during the progression of treatment. The standard drug, MET, exhibited the most pronounced antihyperglycemic effect (Figure 6A). Nevertheless, the damage to pancreatic β-cells after the administration of STZ led to decreased glucose tolerance and resistance to insulin, which increased blood glucose levels in the DC group (Zhang et al., 2019).

After the study was completed, an OGTT was conducted, and the AUC is depicted in Figure 6C F. Before orally administering glucose (0 min), the FBG levels of the DC group rats were notably elevated (p < 0.001) in comparison to the other groups. Following oral glucose administration, all groups exhibited a rapid elevation in blood glucose levels at 15 and 30 min. However, after 30 min, the blood glucose levels of rats in the bioactive fraction 8 and MET-treated groups began to decrease significantly (p < 0.001) in comparison with those in the DC group. The DC group rats demonstrated a rapid elevation (p < 0.001) in FBG after oral glucose administration, compared to the NC group, which persisted for 120 min, suggesting that the DC group had decreased glucose tolerance. Furthermore, the AUC derived from the OGTT was notably elevated in the DC group compared to the NC group, demonstrating significant impairment in managing externally administered glucose (Varga et al., 2021). Treatment with bioactive fraction 8 for 28 days exhibited a remarkable and substantial improvement in impaired glucose tolerance (p < 0.05) compared to the MET-treated group. F8-H and MET showed comparable efficacy in improving glucose intolerance in diabetic rats during the OGTT (Figure 6B). These findings suggest that the isolated bioactive fraction could aid T2DM rats in maintaining normal glucose metabolism.

### 3.9 Effect of bioactive fraction 8 on FINS and HOMA-IR

**FIGURE 7:**
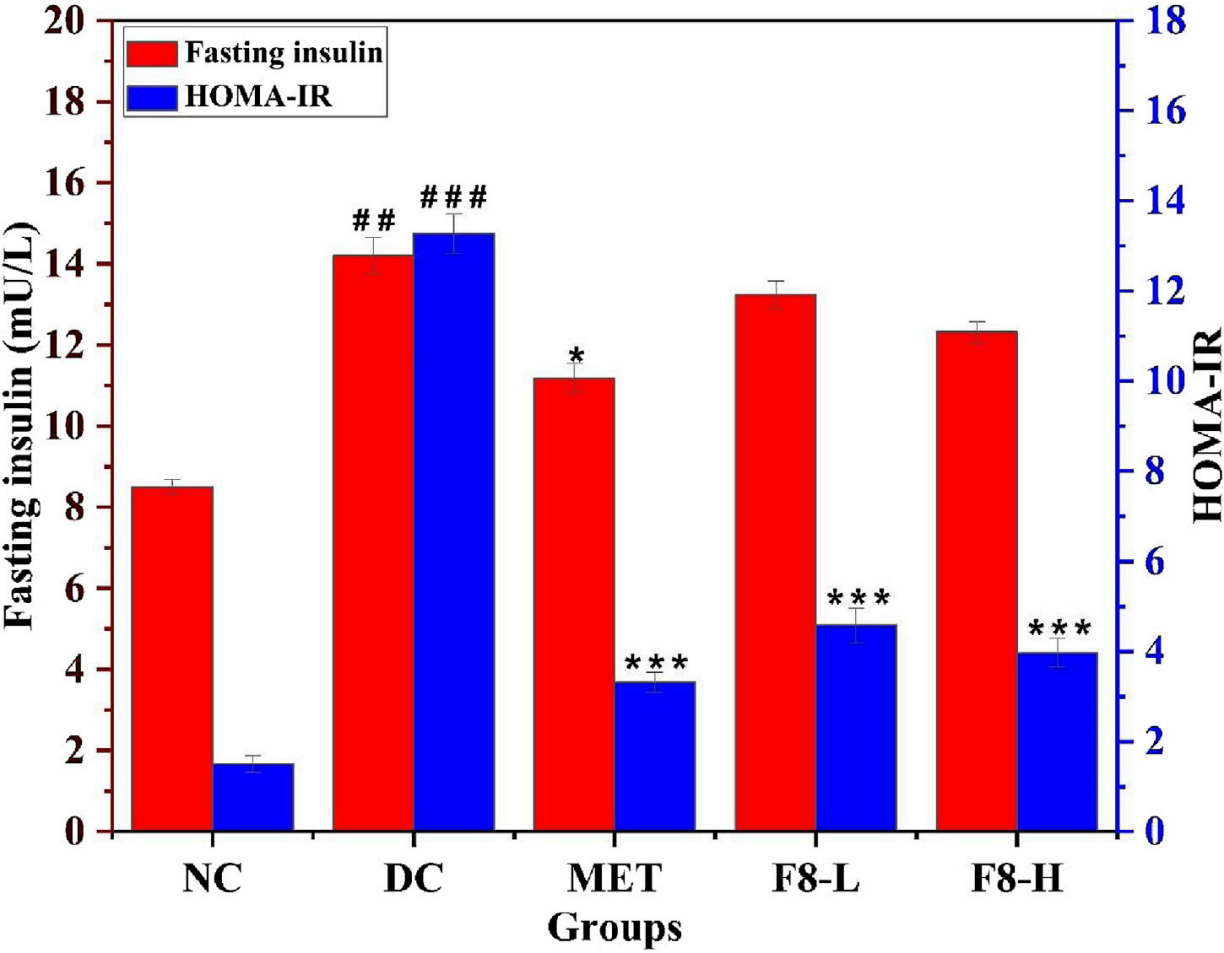
Effect of bioactive fraction 8 on FINS level and HOMA-IR. The values are represented as the mean ± standard deviation (n = 6). Results were analysed using one-way ANOVA, and significant differences were denoted as ^###^p < 0.001, ^##^p < 0.01 in comparison with the NC group, whereas ***p < 0.001, *p < 0.05 in comparison with the DC group.

The impact of bioactive fraction 8 on FINS was investigated, and the HOMA-IR score was calculated to evaluate insulin sensitivity. FINS and HOMA-IR scores were substantially elevated in diabetic groups (14.21 ± 0.44 mU/L and 13.27 ± 0.44) in comparison to the NC group (8.5 ± 0.19 mU/L and 1.51 ± 0.19), indicating substantial insulin resistance. After 28 days of treatment with bioactive fraction 8, both FINS levels and HOMA-IR scores were notably reduced (p < 0.001) compared to the diabetic rats, suggesting that the isolated bioactive fraction and MET improved insulin resistance (Figure 7). Jiang and Zhang (2022) previously reported that treatment with nerolidol reduced HOMA-IR levels in diabetic rats. Bioactive fraction 8, particularly F8-H treated groups, exhibited enhanced control over FBG, OGTT, and FINS levels (p < 0.05) and the findings closely mirrored those of the standard group MET. After 28 days of treatment, enhanced insulin sensitivity was seen in the F8-L, F8-H, and MET-treated groups. A previous study reported that treatment with nerolidol significantly reduced FBG and FINS levels in rat models fed with a diet excessive in calories (Sabir et al., 2022). The regulated levels of FBG, OGTT, and FINS improved insulin sensitivity and contributed to regulating blood glucose levels (Wang et al., 2019). Therefore, F8-H could be further evaluated and utilised for T2DM management.

### 3.10 Effect of bioactive fraction 8 on lipid profile

**FIGURE 8:**
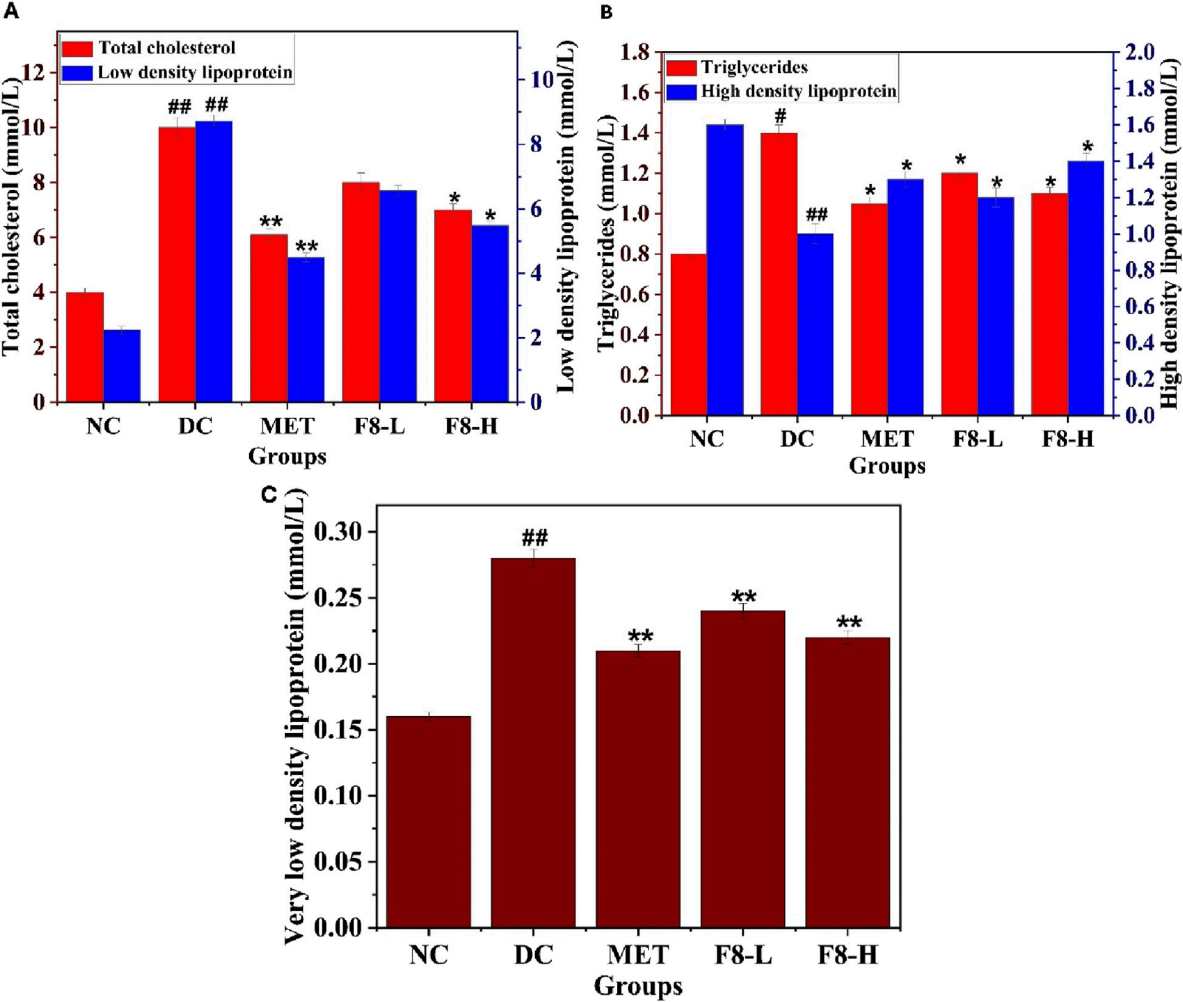
Effect of bioactive fraction 8 on **(A)** Total cholesterol, Low-density lipoprotein, **(B)** Triglycerides, High-density lipoprotein, **(C)** Very low-density lipoprotein. The values are represented as the mean ± standard deviation (n = 6). Results were analysed using one-way ANOVA, and significant differences were denoted as ^##^p < 0.01, ^#^p < 0.05 in comparison with the NC group, whereas **p < 0.01, *p < 0.05 in comparison with the DC group.

The impact of bioactive fraction 8 on TC, TG, HDL, LDL, and VLDL levels was investigated after 28 days of treatment since dyslipidemia is linked to insulin resistance. The enhanced levels of TC, TG, LDL, and VLDL were substantially decreased (p < 0.05), and conversely, HDL was increased (p < 0.05) in the F8-L, F8-H, and MET-treated groups in comparison with the diabetic group (Figures 8A–C). It was previously reported that treatment with nerolidol decreased TC, TG, LDL, and VLDL levels and increased HDL levels in diabetic rats (Jiang and Zhang, 2022). The results of the lipid profile test indicate that the isolated bioactive fraction has significant antihyperlipidemic effects compared to the standard drug, MET-treated group (p < 0.05). The AI decreased substantially in the F8-L (p < 0.01), F8-H (p < 0.001), and MET (p < 0.001) treated groups compared to the DC group (Table 6), suggesting that isolated bioactive fraction 8 may decrease the risk for cardiovascular diseases. Nevertheless, lipid and carbohydrate metabolism are interconnected, and abnormalities in these processes are linked to cardiovascular disorders (Ranganathan and Mahalingam, 2020).

**TABLE 6:** Atherogenic index of bioactive fraction 8.

Groups	Atherogenic index
NC	1.5 ± 1.12
DC^###^	9 ± 3.54
MET**	3.36 ± 2.74
F8-L*	5.67 ± 2.86
F8-H**	4.38 ± 1.85

The values are represented as the mean ± standard deviation (n = 6). Results were analysed using one-way ANOVA, and significant differences were denoted as^###^p < 0.001 in comparison with the NC, group, whereas **p < 0.01, *p < 0.05 in comparison with the DC, group.

### 3.11 Effect of bioactive fraction 8 on liver profile

**FIGURE 9:**
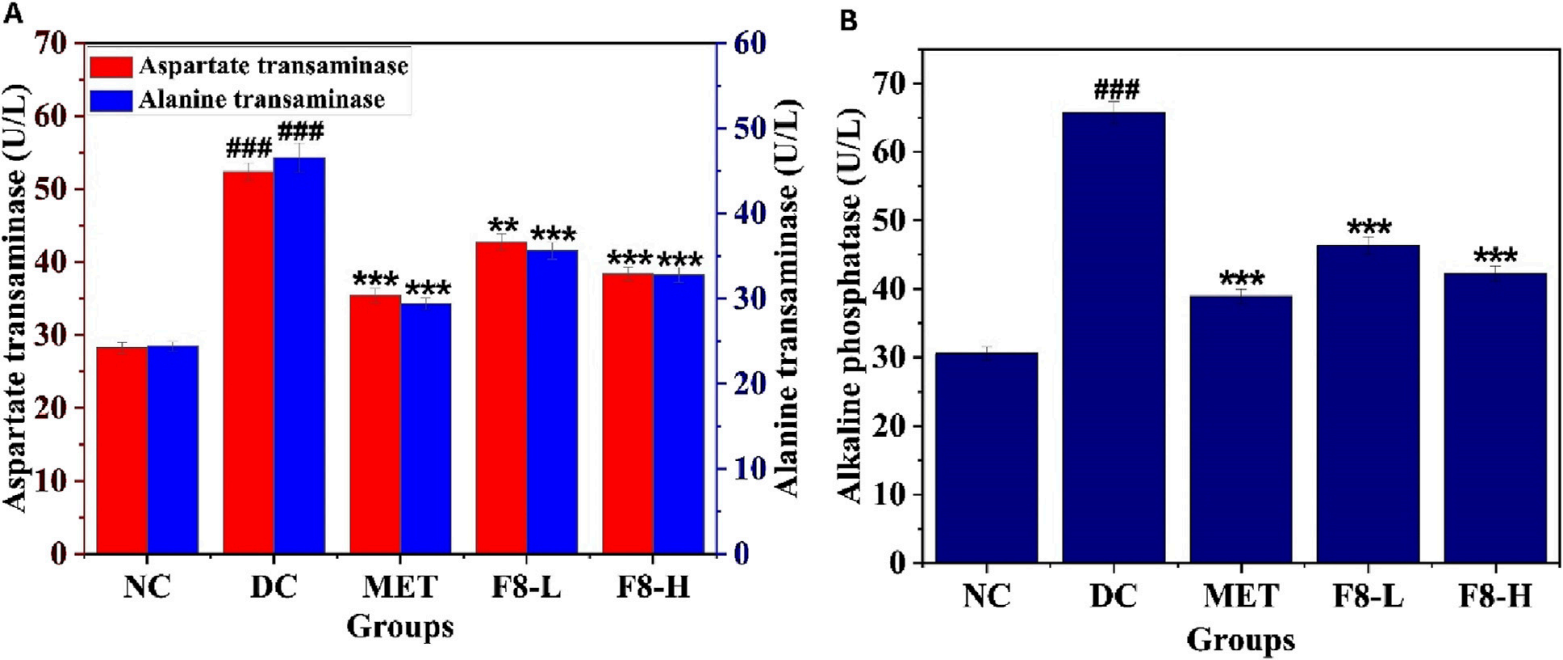
Effects of bioactive fraction 8 on **(A)** AST, ALT, **(B)** ALP. The values are represented as the mean ± standard deviation (n = 6). Results were analysed using one-way ANOVA, and significant differences were denoted as ^###^p < 0.001 compared to the NC group, whereas ***p < 0.001, **p < 0.01 compared to the DC group.

The impact of bioactive fraction 8 on liver enzymes AST, ALP, and ALT, which serve as biomarkers for assessing liver function, was studied. Hepatic and glucose metabolism are interconnected; thus, diabetes mellitus leads to irregularities in liver metabolism (Saltiel and Kahn, 2001). AST and ALT are mainly present in liver cells, and their release into the circulatory system signifies impaired liver function owing to irregular glucose metabolism and impaired hepatic tissue. Therefore, the liver markers AST, ALP, and ALT indicate hepatic impairment resulting from diabetes mellitus (Chen et al., 2017). The AST, ALT, and ALP levels were substantially elevated in the diabetic groups. Following 28 days of treatment, an alleviating effect was observed in the F8-L, F8-H, and MET-treated groups. (Jiang and Zhang, 2022). also reported that treatment with nerolidol improved AST, ALT, and ALP levels in diabetic rats. (Sabir et al., 2022). reported that treatment with nerolidol decreased lipid accumulation in the liver caused by a diet excessive in calories. The impact of F8-H closely resembled that of the standard drug, MET (p < 0.05), as depicted in Figures 9A,B. The liver profile test demonstrated a significant reduction in liver damage resulting from STZ-induced diabetes mellitus following treatment with the isolated bioactive fraction.

### 3.12 Effect of bioactive fraction 8 on kidney profile

**FIGURE 10:**
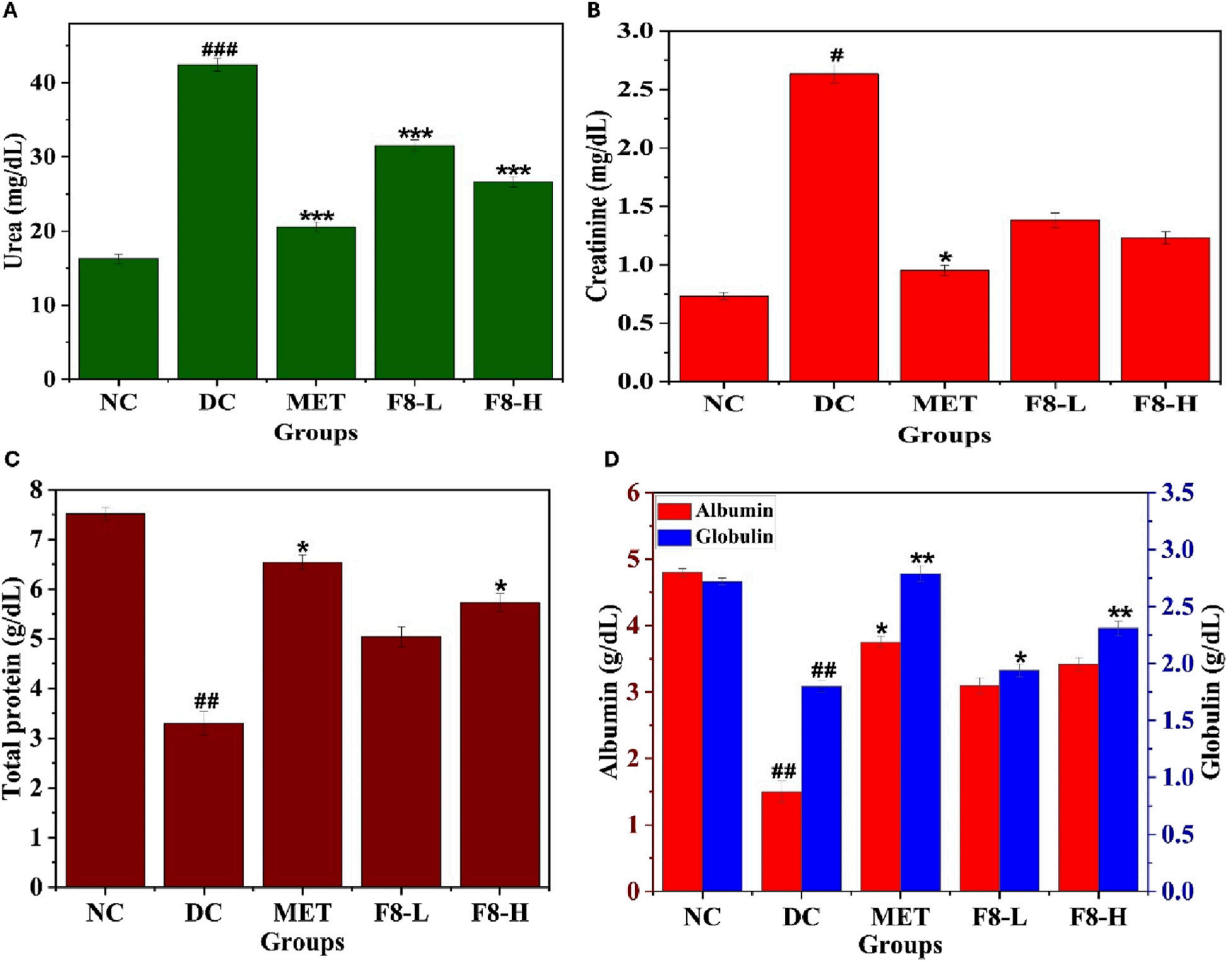
Effects of bioactive fraction 8 on **(A)** Urea, **(B)** Creatinine, **(C)** Total protein, **(D)** Albumin, Globulin. The values are represented as the mean ± standard deviation (n = 6). Results were analysed using one-way ANOVA, and significant differences were denoted as ^###^p < 0.001, ^##^p < 0.01, ^#^p < 0.05 compared to the NC group, whereas ***p < 0.001, **p < 0.01, *p < 0.05 compared to the DC group.

The impact of bioactive fraction 8 on kidney biomarkers, including creatinine, urea, total protein, globulin, and albumin, was tested. The DC group exhibited higher levels of urea and creatinine than the NC group, while the contrary effect was noticed in the bioactive fraction and MET-treated groups, as shown in Figures 10A,B. The DC group also exhibited lower levels of total protein, albumin, and globulin compared to the NC group, while the contrary effect was noticed in the bioactive fraction 8 and MET-treated groups (Figures 10C,D). Nevertheless, diabetic kidney disease is a critical complication of T2DM (Xu et al., 2018), and the results of kidney profile tests suggest that F8-L and F8-H ameliorate kidney function (p < 0.05) compared to the MET-treated group. Analysis of biochemical parameters indicates that F8-H demonstrated improved control over FBG, FINS, OGTT levels, and lipid, liver, and kidney profiles. Hence, F8-H can be used to manage T2DM.

### 3.13 Histopathological studies of isolated bioactive fraction 8

#### 3.13.1 Effect of bioactive fraction 8 on liver

**FIGURE 11:**
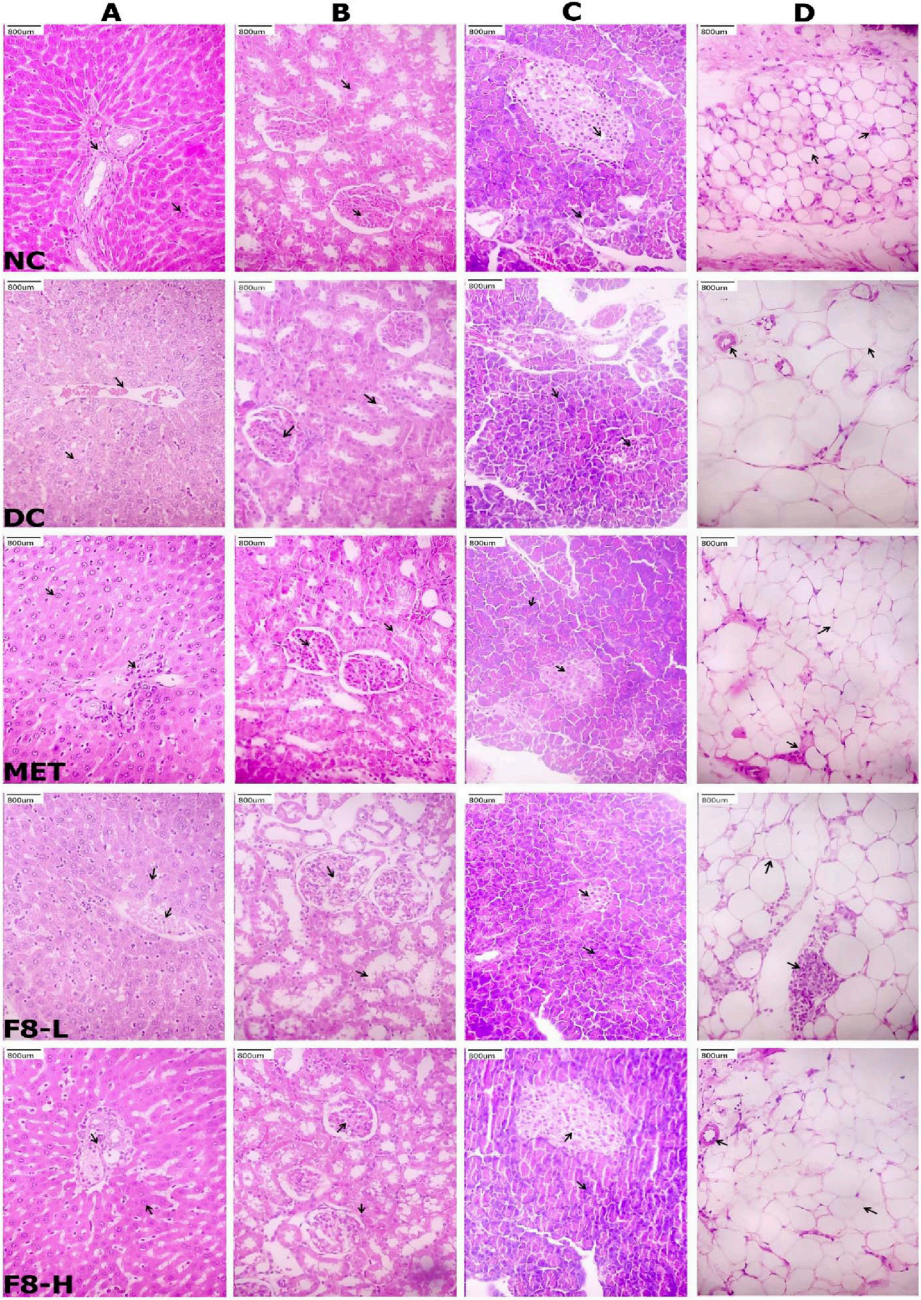
Effects of bioactive fraction 8 on the histology of **(A)** liver, **(B)** kidney, **(C)** pancreas, and **(D)** epididymal adipose tissue (magnification, ×800)

F8-L and F8-H treated groups displayed normal liver parenchyma and portal tubules, closely related to the MET-treated groups (Figure 11A). Portal tubules in the diabetic groups exhibited moderate hyperplasia of the bile duct, congestion, and inflammation, potentially attributed to STZ induction. The NC group showed the typical architecture of liver parenchyma with integral and continuously arranged hepatocytes. Analysis of liver tissue indicates that the isolated bioactive fraction does not harm liver parenchyma cells.

#### 3.13.2 Effect of bioactive fraction 8 on kidney

The NC, F8-L, F8-H, and MET-treated groups displayed normal tubules, glomerulus, interstitium, and blood vessels (Figure 11B). Histopathological analysis showed that the isolated bioactive fraction has no toxic effects on kidney cells. The DC group exhibited substantial impairment in the kidney tissue, characterised by endocapillary proliferation in glomerular structures and hydropic deterioration of the renal tubules, along with mild edema in the interstitial spaces.

#### 3.13.3 Effect of bioactive fraction 8 on pancreas

The pancreatic islet cells were eliminated, and the normal pancreatic acini were observed in the DC group of rats. This shows that the STZ injected specifically targeted and eliminated the pancreatic β-cells. NC group rats showed normal pancreatic islet cells and pancreatic acini. The F8-L, F8-H, and MET-treated groups exhibited signs of regeneration of normal pancreatic parenchyma, like the NC group rats; specifically, the quantity of pancreatic islet cells increased, a gradual improvement in their shape, and a more apparent peripheral tissue structure (Figure 11C).

#### 3.13.4 Effect of bioactive fraction 8 on epididymal adipose tissue

The histological analysis of epididymal adipose tissue revealed that DC group rats had enlarged and irregularly shaped adipocytes compared to the NC group, where the adipocytes were smaller and densely packed. The enlarged adipocytes of diabetic rats were improved following 28 days of treatment with F8-L, F8-H, and MET (Figure 11D). Treatment with F8-H and MET significantly decreased the size of adipocytes in comparison with the DC group. The results indicate that F8-H could potentially alleviate dyslipidemia and enhance insulin sensitivity. Histopathological analysis indicates that F8-H has favourable impacts on the histology of the liver, kidney, pancreas, and epididymal adipose tissue. Therefore, F8-H can be used to manage T2DM.

### 3.14 Profiling of gene expression

**FIGURE 12:**
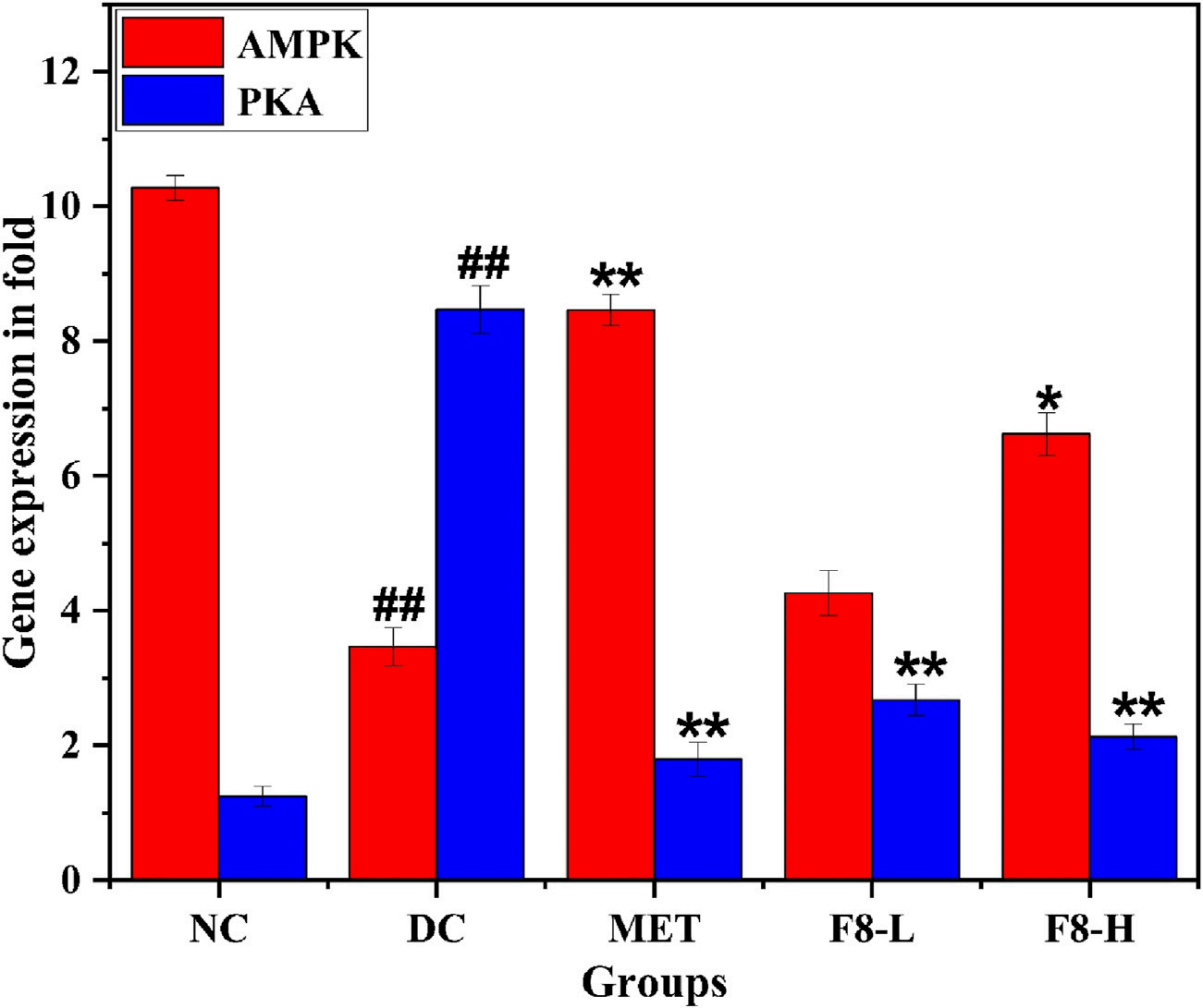
Expression of AMPK and PKA genes in fold. The values are represented as the mean ± standard deviation (n = 3). Results were analysed using one-way ANOVA, and significant differences were denoted as ^# #^p < 0.01 compared to the NC group, whereas **p < 0.01, *p < 0.05 compared to the DC group.

**FIGURE 13:**
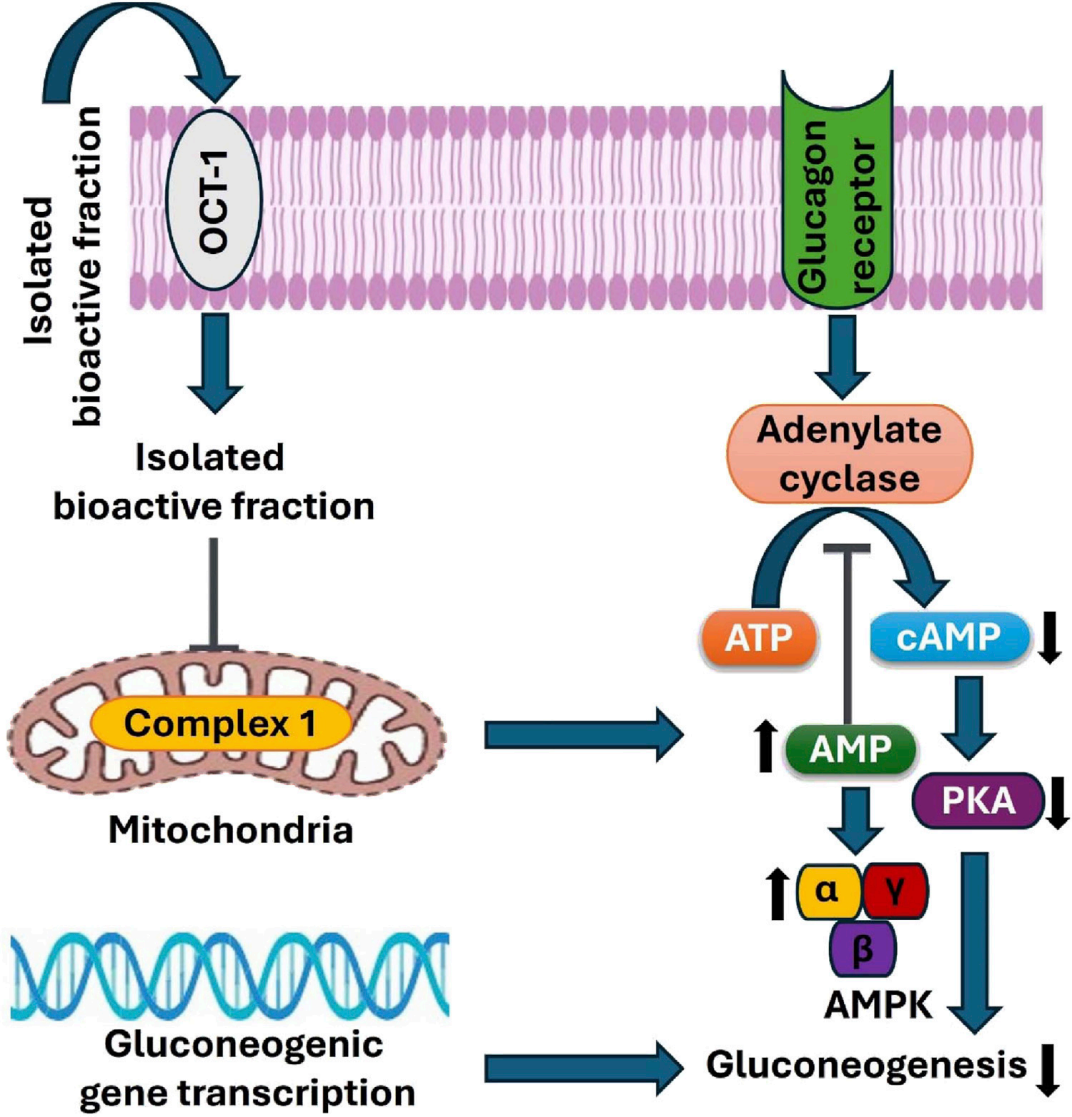
Proposed mechanisms underlying the antidiabetic effects of the bioactive fraction isolated from *Curcuma angustifolia* rhizome in type 2 diabetic rats.

The expression levels of genes of interest, including AMPK and PKA and reference gene β-actin, were measured. After normalising the Ct (threshold cycle) values with the reference gene β-actin, the effect of F8-L and F8-H on AMPK and PKA was assessed. The fold expression values for the transcriptional expressions of AMPK and PKA genes were 4.26 ± 0.33 and 2.68 ± 0.23 for the F8-L treated group, while only the F8-H treated group had significant (P ≤ 0.05) fold expression values of 6.62 ± 0.31 and 2.13 ± 0.19 compared to the diabetic control group (3.47 ± 0.28, 8.47 ± 0.35). F8-H expression values corresponded to the standard drug metformin-treated group (8.46 ± 0.23, 1.8 ± 0.25). The normal control group exhibited fold expression values of 10.28 ± 0.18 for AMPK and 1.25 ± 0.15 for PKA (Figure 12). RT-PCR results indicated that AMPK mRNA levels were significantly higher in metformin, F8-L, and F8-H treated groups, with a corresponding reduction in the PKA gene expression compared to the diabetic control group. As depicted in Figure 12, the AMPK gene was found to be upregulated, and the PKA gene was found to be downregulated. The increased expression of the PKA gene in the diabetic group may be attributed to an increase in glucagon levels. A high glucagon level is generally observed in T2DM. It is crucial in activating PKA via cAMP, leading to hepatic glucose production through the gluconeogenesis pathway (Ranganathan and Mahalingam, 2020). Glucagon utilises the adenylate cyclase to convert ATP to cAMP, activating PKA. PKA has a critical role in controlling the transcription of genes related to gluconeogenesis, which enhances the production of hepatic glucose by impeding glycolysis (Gao et al., 2021). The AMPK gene is expressed in the F8-L, F8-H, and metformin-treated groups, which may be attributed to the inhibition of mitochondrial complex-1. Nevertheless, mitochondrial complex-1 is the direct target of metformin (Feng et al., 2022). Therefore, the mechanism of action of the isolated bioactive fraction may be similar to that of the standard drug metformin, as depicted in Figure 13. Inhibition of mitochondrial complex-1 decreases the production of ATP. It elevates the cytoplasm’s ADP level, which activates AMPK (Agius et al., 2020). When AMPK is activated, it prevents adenylate cyclase activity, stopping the impact of cAMP. This results in the halting of gluconeogenesis activated by PKA and other transcription processes regulated by PKA (Jayarajan et al., 2019). Additionally, glucose is the product of PKA expression, which can be estimated by measuring blood glucose levels. Blood glucose levels were significantly reduced after 28 days of F8-L and F8-H treatment, as shown in Figure 6A. After 28 days of treatment with isolated bioactive fraction, both FINS levels and HOMA-IR scores were also notably reduced, suggesting that the bioactive fraction improved insulin resistance (Figure 7). Bioactive fraction 8, particularly F8-H treated groups, exhibited enhanced control over FBG, OGTT, and FINS levels. The regulated levels of FBG, OGTT, FINS, and HOMA-IR scores improved insulin sensitivity and contributed to regulating blood glucose levels (Wang et al., 2019). The regulated blood glucose levels may be attributed to the upregulation of AMPK and the corresponding downregulation of PKA. The mechanism of action of the isolated bioactive fraction may be corresponding to metformin.

## 4 Conclusion

Bioactive fraction isolated from methanolic extract of *C. angustifolia* rhizome ameliorated hyperglycemia *in vitro* and *in vivo*. The isolated bioactive fraction inhibited α-amylase and α-glucosidase enzymes *in vitro* significantlycompared to the standard drug, acarbose. Molecular docking analysis also showed that nerolidol, the major compound of the isolated bioactive fraction has competitive inhibitory effects against α-amylase and α-glucosidase enzymes. According to the *in silico* ADMET predictions, nerolidol has the optimal physicochemical characteristics for drug-likeness. Bioactive fraction significantly improved hyperglycemia and hyperlipidemia in type 2 diabetic rats caused by HFD and a low dose of STZ, as shown by a significant decrease in body weight loss, FBG level, enhancing glucose tolerance, regulated lipid metabolism, and mitigated insulin resistance *in vivo*. Compared to metformin, F8-H showed a significantly ameliorating effect on liver and kidney profiles than F8-L. Histopathological analysis demonstrated that pancreatic β-cells were regenerated in all treated groups (F8-L, F8-H, and metformin). Furthermore, F8-H had a more significant impact on the histology of the liver, kidney, pancreas, and epididymal adipose tissue compared to F8-L. The underlying mechanism is associated with the upregulation of the AMPK pathway by downregulating the PKA pathway, similar to the standard drug metformin. Thus, this study also revealed that treatment with the isolated bioactive fraction could regulate hepatic glucose metabolism in type 2 diabetic rats, inhibiting gluconeogenesis. The levels of controlled FBG were determined by activating AMPK. Inhibiting the over-expression of gluconeogenesis and lipogenesis through upregulation of the AMPK pathway effectively regulated glucolipid homeostasis and improved diabetes. The findings of this study indicate that the isolated bioactive fraction is significantly effective in diabetes management, addressing associated complications such as insulin resistance, glucose intolerance, and hyperlipidemia, and can be explored as a natural alternative to synthetic medications commonly used for treating T2DM.

## Data Availability

The original contributions presented in the study are included in the article/Supplementary Material, further inquiries can be directed to the corresponding author.

## Glossary

ADMET: Absorption, Distribution, Metabolism, Excretion, and Toxicity
AMPK: Adenosine monophosphate-activated protein kinase
ALT: Alanine transaminase
ALP: Alkaline phosphatase
AUC: Area under the curve
AST: Aspartate transaminase
AI: Atherogenic index
CPCSEA: Committee for the Purpose of Control and Supervision of Experiments on Animals
cDNA: Complementary DNA
*C. angustifolia*: *Curcuma angustifolia*
cAMP: Cyclic AMP
CYP: Cytochrome P450
DC: Diabetic control
MTT: 3-[4,5-dimethylthiazol-2-yl]-2,5 diphenyl tetrazolium bromide
FBG: Fasting blood glucose
FINS: Fasting insulin
FTIR: Fourier transform infrared spectroscopy
GC-MS: Gas chromatography-mass spectrometry
**IC**_**50**_: Half maximal inhibitory concentration
HDL-c: High-density lipoprotein cholesterol
HFD: High-fat diet
F8-H: Higher dose of fraction 8
HepG2: Human hepatoma
HOMA-IR: Insulin sensitivity resistance index
LDL: Low-density lipoprotein
F8-L: Lower dose of fraction 8
**LD**_**50**_: Median lethal dose
mRNA: Messenger RNA
MET: Metformin
NIST: National Institute of Standards and Technology
NC: Normal control
OGTT: Oral glucose tolerance test
PBS: Phosphate-buffered saline
PKA: Protein kinase A
^**1**^ **H NMR**: Proton nuclear magnetic resonance
qRT-PCR: Quantitative real-time polymerase chain reaction
**R**_**f**_: Retention factor
RNA: Ribonucleic acid
STZ: Streptozotocin
TLC: Thin layer chromatography
Ct: Threshold cycle
TC: Total cholesterol
TG: Triglycerides
T2DM: Type 2 diabetes mellitus
VLDL: Very low-density lipoprotein

## References

[B1] Agius L. Ford B. E. Chachra S. S. (2020). The metformin mechanism on gluconeogenesis and AMPK activation: the metabolite perspective. Int. J. Mol. Sci. 21, 3240. 10.3390/ijms21093240 32375255 PMC7247334

[B2] Akhtar M. T. Almas T. Safdar S. Saadia M. Qadir R. Batool S. (2023). Antioxidant, hypoglycemic, antilipidemic, and protective effect of polyherbal emulsion (F6-SMONSECCE) on alloxan-induced diabetic rats. ACS Omega 8, 21642–21652. 10.1021/acsomega.3c01027 37360421 PMC10286276

[B3] Alhamhoom Y. Ahmed S. S. Salahuddin M. D. D R. B. Ahmed M. M. Farhana S. A. (2023). Synergistic antihyperglycemic and antihyperlipidemic effect of polyherbal and allopolyherbal formulation. Pharmaceuticals 16, 1368. 10.3390/ph16101368 37895839 PMC10610467

[B4] Banks W. A. Rhea E. M. (2021). The blood – brain barrier, oxidative stress, and insulin resistance. Antioxidants 10, 1695. 10.3390/antiox10111695 34829566 PMC8615183

[B5] Batiha G. E.-S. Alqahtani A. Ojo O. A. Shaheen H. M. Wasef L. Elzeiny M. (2020). Biological properties, bioactive constituents, and pharmacokinetics of some capsicum spp. and capsaicinoids. Int. J. Mol. Sci. 21, 5179. 10.3390/ijms21155179 32707790 PMC7432674

[B6] Biondo L. A. Batatinha H. A. Souza C. O. Teixeira A. A. S. Silveira L. S. Alonso-Vale M. I. (2018). Metformin mitigates fibrosis and glucose intolerance induced by doxorubicin in subcutaneous adipose tissue. Front. Pharmacol. 9, 452. 10.3389/fphar.2018.00452 29867463 PMC5952005

[B7] Carrasco-Correa E. J. Ruiz-Allica J. Rodríguez-Fernández J. F. Miró M. (2021). Human artificial membranes in (bio)analytical science: potential for *in vitro* prediction of intestinal absorption-A review. Trac. Trends Anal. Chem. 145, 116446. 10.1016/j.trac.2021.116446

[B8] Chen S. C.-C. Tsai S. P. Jhao J.-Y. Jiang W.-K. Tsao C. K. Chang L.-Y. (2017). Liver fat, hepatic enzymes, alkaline phosphatase and the risk of incident type 2 diabetes: a prospective study of 132,377 adults. Sci. Rep. 7, 4649. 10.1038/s41598-017-04631-7 28680048 PMC5498613

[B9] Dai Y. Shen Y. Guo J. Yang H. Chen F. Zhang W. (2024). Glycolysis and gluconeogenesis are involved of glucose metabolism adaptation during fasting and re-feeding in black carp (*Mylopharyngodon piceus*). Aquac. Fish. 9, 226–233. 10.1016/j.aaf.2022.04.003

[B10] Daoui O. Elkhattabi S. Chtita S. (2022). Rational design of novel pyridine-based drugs candidates for lymphoma therapy. J. Mol. Struct. 1270, 133964. 10.1016/j.molstruc.2022.133964

[B11] Fajriaty I. Fidrianny I. Kurniati N. F. Fauzi N. M. Mustafa S. H. Adnyana I. K. (2024). *In vitro* and *in silico* studies of the potential cytotoxic, antioxidant, and HMG CoA reductase inhibitory effects of chitin from Indonesia mangrove crab (*Scylla serrata*) shells. Saudi J. Biol. Sci. 31, 103964. 10.1016/j.sjbs.2024.103964 38500815 PMC10945265

[B12] Feng J. Wang X. Ye X. Ares I. Lopez-Torres B. Martínez M. (2022). Mitochondria as an important target of metformin: the mechanism of action, toxic and side effects, and new therapeutic applications. Pharmacol. Res. 177, 106114. 10.1016/j.phrs.2022.106114 35124206

[B13] Gao M. Deng C. Dang F. (2021). Synergistic antitumor effect of resveratrol and sorafenib on hepatocellular carcinoma through PKA/AMPK/eEF2K pathway. Food Nutr. Res. 65. 10.29219/fnr.v65.3602 PMC8559449 34776832

[B14] Ghasemi M. Turnbull T. Sebastian S. Kempson I. (2021). The MTT assay: utility, limitations, pitfalls, and interpretation in bulk and single-cell analysis. Int. J. Mol. Sci. 22, 12827. 10.3390/ijms222312827 34884632 PMC8657538

[B15] Gök H. N. Deliorman Orhan D. Gürbüz İ. Aslan M. (2020). Activity-guided isolation of α-amylase, α-glucosidase, and pancreatic lipase inhibitory compounds from Rhus coriaria L. J. Food Sci. 85, 3220–3228. 10.1111/1750-3841.15438 32895959

[B16] Gonfa T. Fisseha A. Thangamani A. (2020). Isolation, characterization and drug-likeness analysis of bioactive compounds from stem bark of Warburgia ugandensis Sprague. Chem. Data Collect. 29, 100535. 10.1016/j.cdc.2020.100535

[B17] Guo T. Yan W. Cui X. Liu N. Wei X. Sun Y. (2023). Liraglutide attenuates type 2 diabetes mellitus - associated non - alcoholic fatty liver disease by activating AMPK/ACC signaling and inhibiting ferroptosis. Mol. Med. 29, 132. 10.1186/s10020-023-00721-7 37770820 PMC10540362

[B18] Hafeez S. Ahmad M. Ishtiaq S. Ajaib M. Husnain S. Shahwar D. (2024). Metabolite profiling and biochemical investigation of the antidiabetic potential of Loranthus pulverulentus Wall n -butanol fraction in diabetic animal models. J. Ethnopharmacol. 318, 116963. 10.1016/j.jep.2023.116963 37495027

[B19] Harding J. L. Weber M. B. Shaw J. E. (2024). “The global burden of diabetes,” in Textbook of diabetes (John Wiley and Sons, Ltd), 28–40.

[B20] Hassan M. Rasul A. Ajmal Shah M. Jabeen F. Sadiqa A. (2023). Effect of PENN-DIABEX, a novel polyherbal formulation, in high fat diet streptozotocin-induced diabetic rats. Saudi J. Biol. Sci. 30, 103816. 10.1016/j.sjbs.2023.103816 37841663 PMC10568417

[B21] Ilic I. Ilic M. (2024). The burden of type 2 diabetes mellitus in Latin America, 1990–2019: findings from the Global Burden of Disease study. Public Health 233, 74–82. 10.1016/j.puhe.2024.05.009 38852206

[B22] Janzen N. R. Whitfield J. Hoffman N. J. (2018). Interactive roles for AMPK and glycogen from cellular energy sensing to exercise metabolism. Int. J. Mol. Sci. 19, 3344. 10.3390/ijms19113344 30373152 PMC6274970

[B23] Jayarajan V. Appukuttan A. Aslam M. Reusch P. Regitz-Zagrosek V. Ladilov Y. (2019). Regulation of AMPK activity by type 10 adenylyl cyclase: contribution to the mitochondrial biology, cellular redox and energy homeostasis. Cell. Mol. Life Sci. 76, 4945–4959. 10.1007/s00018-019-03152-y 31172217 PMC11105217

[B24] Jia C.-Y. Li J.-Y. Hao G.-F. Yang G.-F. (2020). A drug-likeness toolbox facilitates ADMET study in drug discovery. Drug Discov. Today 25, 248–258. 10.1016/j.drudis.2019.10.014 31705979

[B25] Jiang N. Zhang Y. (2022). Antidiabetic effects of nerolidol through promoting insulin receptor signaling in high-fat diet and low dose streptozotocin-induced type 2 diabetic rats. Hum. \and Exp. Toxicol. 41, 09603271221126487. 10.1177/09603271221126487 36169646

[B26] Kaewin S. Poolsri W. Korkut G. G. Patrakka J. Aiebchun T. Rungrotmongkol T. (2023). A sulfonamide chalcone AMPK activator ameliorates hyperglycemia and diabetic nephropathy in db/db mice. Biomed. Pharmacother. 165, 115158. 10.1016/j.biopha.2023.115158 37473685

[B27] Kato-Schwartz C. G. Corrêa R. C. G. de Souza Lima D. de Sá-Nakanishi A. B. de Almeida Gonçalves G. Seixas F. A. V. (2020). Potential anti-diabetic properties of Merlot grape pomace extract: an *in vitro*, *in silico* and *in vivo* study of α-amylase and α-glucosidase inhibition. Food Res. Int. 137, 109462. 10.1016/j.foodres.2020.109462 33233136

[B28] Kavya P. Gayathri M. (2024). Phytochemical profiling and assessment of antidiabetic activity of Curcuma angustifolia rhizome methanolic extract: an *in vitro* and *in silico* analysis. Chem. and Biodivers. 21, e202301788. 10.1002/cbdv.202301788 38484132

[B29] Kavya P. Theijeswini R. C. Gayathri M. (2024). Phytochemical analysis, identification of bioactive compounds using GC-MS, *in vitro* and *in silico* hypoglycemic potential, *in vitro* antioxidant potential, and *in silico* ADME analysis of Chlorophytum comosum root and leaf. Front. Chem. 12, 1458505–1458525. 10.3389/fchem.2024.1458505 39345858 PMC11427758

[B30] Kumar A. Negi A. S. Chauhan A. Semwal R. Kumar R. Semwal R. B. (2022). Formulation and evaluation of SGLT2 inhibitory effect of a polyherbal mixture inspired from Ayurvedic system of medicine. J. Tradit. Complement. Med. 12, 477–487. 10.1016/j.jtcme.2022.03.003 36081821 PMC9446025

[B31] Lee J.-M. Seo W.-Y. Song K.-H. Chanda D. Kim Y. D. Kim D.-K. (2010). AMPK-Dependent repression of hepatic gluconeogenesis via disruption of CREB·CRTC2 complex by orphan nuclear receptor small heterodimer partner. J. Biol. Chem. 285, 32182–32191. 10.1074/jbc.M110.134890 20688914 PMC2952219

[B32] Li J. Wei Y. Huang S. Yan S. Zhao B. Wang X. (2024a). Hyperglycemia effect of Pinctada martensii hydrolysate in diabetic db/db mice. J. Ethnopharmacol. 319, 117104. 10.1016/j.jep.2023.117104 37659759

[B33] Li Y. Zhang W. Tang C. Wang C. Liu C. Chen Q. (2024b). Antidiabetic effects and mechanism of γ-polyglutamic acid on type II diabetes mice. Int. J. Biol. Macromol. 261, 129809. 10.1016/j.ijbiomac.2024.129809 38290633

[B34] Lin J. Li M. Mak W. Shi Y. Zhu X. Tang Z. (2022). Applications of *in silico* models to predict drug-induced liver injury. Toxics 10, 788. 10.3390/toxics10120788 36548621 PMC9785299

[B35] McCorvie T. J. Loria P. M. Tu M. Han S. Shrestha L. Froese D. S. (2022). Molecular basis for the regulation of human glycogen synthase by phosphorylation and glucose-6-phosphate. Nat. Struct. Mol. Biol. 29, 628–638. 10.1038/s41594-022-00799-3 35835870 PMC9287172

[B36] Pandeya P. R. Lamichhane G. Lamichhane R. Luo J. Li X.-J. Rhee S. (2022). Antiobesity activity of two polyherbal formulations in high-fat diet-induced obese C57bl/6J mice. Biomed. Res. Int. 2022, 9120259. 10.1155/2022/9120259 35707380 PMC9192239

[B37] Pasri P. Mermillod P. Khempaka S. (2023). Antioxidant properties and cytotoxic effects of selected edible plants in Southeast Asia for further use as phytogenic antioxidant additives. Saudi J. Biol. Sci. 30, 103631. 10.1016/j.sjbs.2023.103631 37101816 PMC10123259

[B38] Petrović A. Madić V. Stojanović G. Zlatanović I. Zlatković B. Vasiljević P. (2024). Antidiabetic effects of polyherbal mixture made of *Centaurium erythraea*, Cichorium intybus and Potentilla erecta. J. Ethnopharmacol. 319, 117032. 10.1016/j.jep.2023.117032 37582477

[B39] Quimque M. T. J. Magsipoc R. J. Y. Llames L. C. J. Flores A. I. G. Garcia K. Y. M. Ratzenböck A. (2022). Polyoxygenated cyclohexenes from uvaria grandiflora with multi-enzyme targeting properties relevant in type 2 diabetes and obesity. ACS Omega 7, 36856–36864. 10.1021/acsomega.2c05544 36278100 PMC9583304

[B40] Ranganathan N. Mahalingam G. (2020). Validation of the antidiabetic potential of isolated and nanoemulsified endophytic fungal metabolite 2,4,6-triphenylaniline through AMPK activation pathway. J. Mol. Liq. 316, 113836. 10.1016/j.molliq.2020.113836

[B41] Rim K.-T. (2020). *In silico* prediction of toxicity and its applications for chemicals at work. Toxicol. Environ. Health Sci. 12, 191–202. 10.1007/s13530-020-00056-4 32421081 PMC7223298

[B42] Roy A. Mahalingam G. (2017). The *in-vitro* antidiabetic activity of Phoenix roebelenii leaf extract. Int. J. Green Pharm. 11, S128–S134. 10.22377/ijgp.v11i01.884

[B43] Sabir U. Irfan H. M. Alamgeer Ullah A. Althobaiti Y. S. Alshehri F. S. (2022). Downregulation of hepatic fat accumulation, inflammation and fibrosis by nerolidol in purpose built western-diet-induced multiple-hit pathogenesis of NASH animal model. Biomed. Pharmacother. 150, 112956. 10.1016/j.biopha.2022.112956 35447548

[B44] Saltiel A. R. Kahn C. R. (2001). Insulin signalling and the regulation of glucose and lipid metabolism. Nature 414, 799–806. 10.1038/414799a 11742412

[B45] Sansenya S. Winyakul C. Nanok K. Phutdhawong W. S. (2020). Synthesis and inhibitory activity of N-acetylpyrrolidine derivatives on α-glucosidase and α-amylase. Res. Pharm. Sci. 15, 14–25. 10.4103/1735-5362.278711 32180813 PMC7053292

[B46] Sim S. W. Weinstein D. A. Lee Y. M. Jun H. S. (2020). Glycogen storage disease type Ib: role of glucose-6- phosphate transporter in cell metabolism and function. FEBS Lett. 594, 3–18. 10.1002/1873-3468.13666 31705665

[B47] Sunil T. Mala V. (2022). Amelioration of hyperglycemia and hyperlipidemia in a high - fat diet - fed mice by supplementation of a developed optimized polyherbal formulation. 3 Biotech. 12, 1–17. 10.1007/s13205-022-03309-w 36060893 PMC9428098

[B48] Suvarna R. Suryakanth V. B. Bakthavatchalam P. Kalthur G. Nayak M. D. Prabhu M. M. (2023). Acute and sub-chronic toxicity of Liberin, an anti-diabetic polyherbal formulation in rats. J. Ayurveda Integr. Med. 14, 100804. 10.1016/j.jaim.2023.100804 37847964 PMC10585375

[B49] Ugheighele S. E. Imafidon K. E. Choudhary M. I. Shakil A. Khan M. Sherwani Z. A. (2020). Anti-urease and cytotoxic activity of 1-Nitro-2-phenylethane and Nerolidol; two major compounds isolated from the seeds of Dennettia tripetala. Med. Chem. Res. 29, 1874–1881. 10.1007/s00044-020-02607-3

[B50] Varga T. V. Liu J. Goldberg R. B. Chen G. Dagogo-Jack S. Lorenzo C. (2021). Predictive utilities of lipid traits, lipoprotein subfractions and other risk factors for incident diabetes: a machine learning approach in the Diabetes Prevention Program. BMJ Open Diabetes Res. 9, e001953. 10.1136/bmjdrc-2020-001953 PMC8016090 33789908

[B51] Wang H. Huang M. Bei W. Yang Y. Song L. Zhang D. (2021). FTZ attenuates liver steatosis and fibrosis in the minipigs with type 2 diabetes by regulating the AMPK signaling pathway. Biomed. Pharmacother. 138, 111532. 10.1016/j.biopha.2021.111532 34311531

[B52] Wang J. Qi Z. Wu Y. Wang A. Liu Q. Zou F. (2023). Discovery of IHMT-MST1-39 as a novel MST1 kinase inhibitor and AMPK activator for the treatment of diabetes mellitus. Signal Transduct. Target. Ther. 8, 143. 10.1038/s41392-023-01352-4 37015918 PMC10073293

[B53] Wang S. Li G. Zuo H. Yang H. Ma L. Feng J. (2019). Association of insulin, C-peptide and blood lipid patterns in patients with impaired glucose regulation. BMC Endocr. Disord. 19, 75. 10.1186/s12902-019-0400-5 31307454 PMC6631747

[B54] Xie Z. Wang X. Luo X. Yan J. Zhang J. Sun R. (2023). Activated AMPK mitigates diabetes-related cognitive dysfunction by inhibiting hippocampal ferroptosis. Biochem. Pharmacol. 207, 115374. 10.1016/j.bcp.2022.115374 36502872

[B55] Xu L. Lin X. Guan M. Liu Y. (2018). Correlation between different stages of diabetic nephropathy and neuropathy in patients with T2DM: a cross-sectional controlled study. Diabetes Ther. 9, 2335–2346. 10.1007/s13300-018-0519-9 30302722 PMC6250626

[B56] Zhang H. Zhu C. Sun Z. Yan X. Wang H. Xu H. (2019). Linderane protects pancreatic β cells from streptozotocin (STZ) -induced oxidative damage. Life Sci. 233, 116732. 10.1016/j.lfs.2019.116732 31394125

