# Supplementary material for: Bioactive fraction isolated from Curcuma angustifolia rhizome exerts anti-diabetic effects in vitro, in silico and in vivo by regulating AMPK/PKA signaling pathway
Source: Front Pharmacol. 2025 May 14;16:1570533. doi: 10.3389/fphar.2025.1570533 (PMC12116452; doi:10.3389/fphar.2025.1570533)
Supplement: Supplementary file 1 [file Supplementaryfile1.docx]

**SUPPORTING INFORMATION**

**Contents Index:**

| **Entry** | **Contents** | **Figure No.** |
| --- | --- | --- |
| 1 | FTIR spectra of bioactive fraction 8 | SI 1 |
| 2 | 2-D interaction plot of the docked nerolidol into the active site of α-amylase A) nerolidol, B) acarbose, and α-glucosidase C) nerolidol, D) acarbose | SI 2 |
| 3 | A) Boiled-egg graphical representation of nerolidol, B) Bioavailability radar of nerolidol, C) Boiled-egg graphical representation of acarbose, D) Bioavailability radar of acarbose predicted using the swissADME tool | SI 3 |


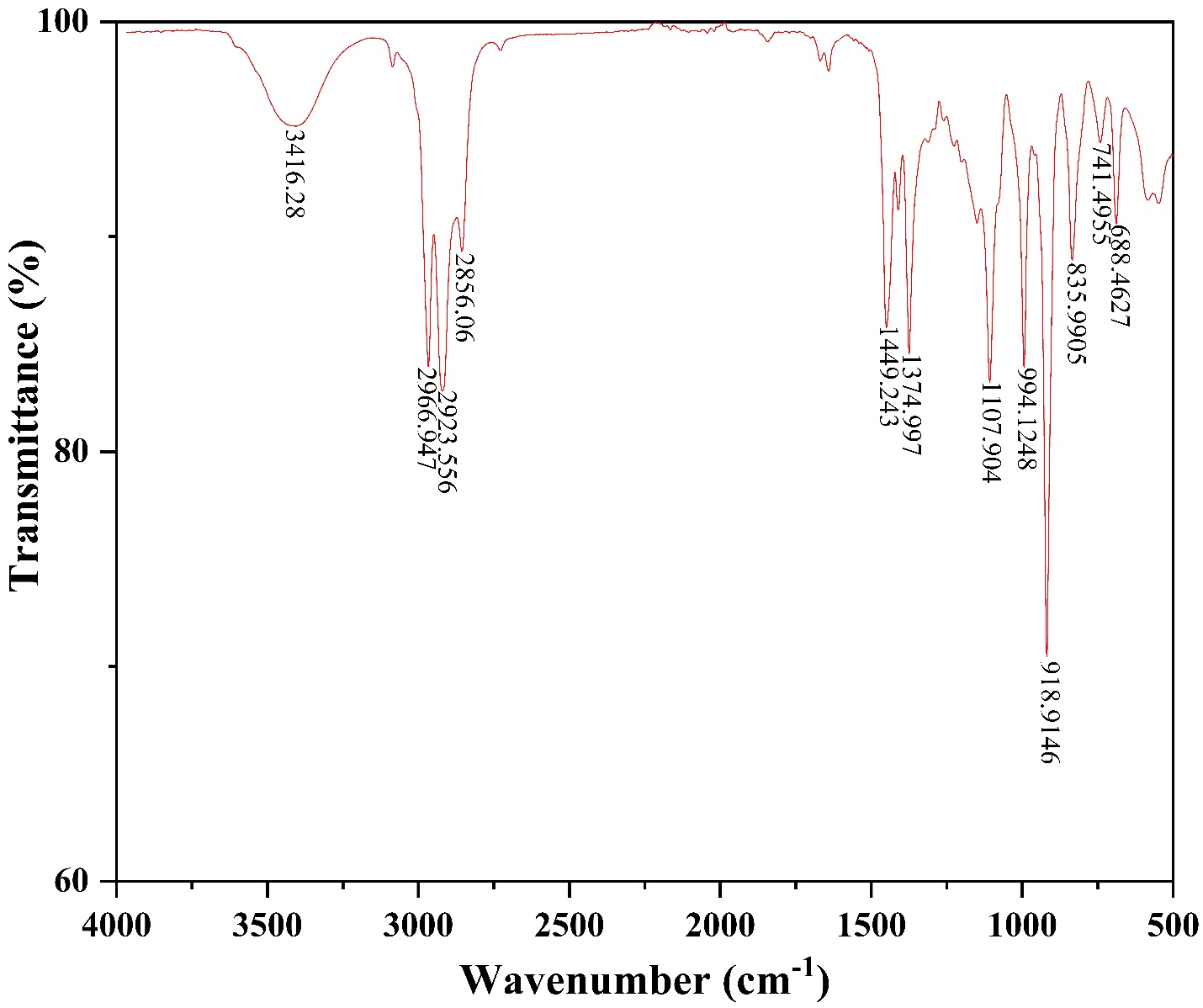


**Fig. SI 1:** FTIR spectra of bioactive fraction 8


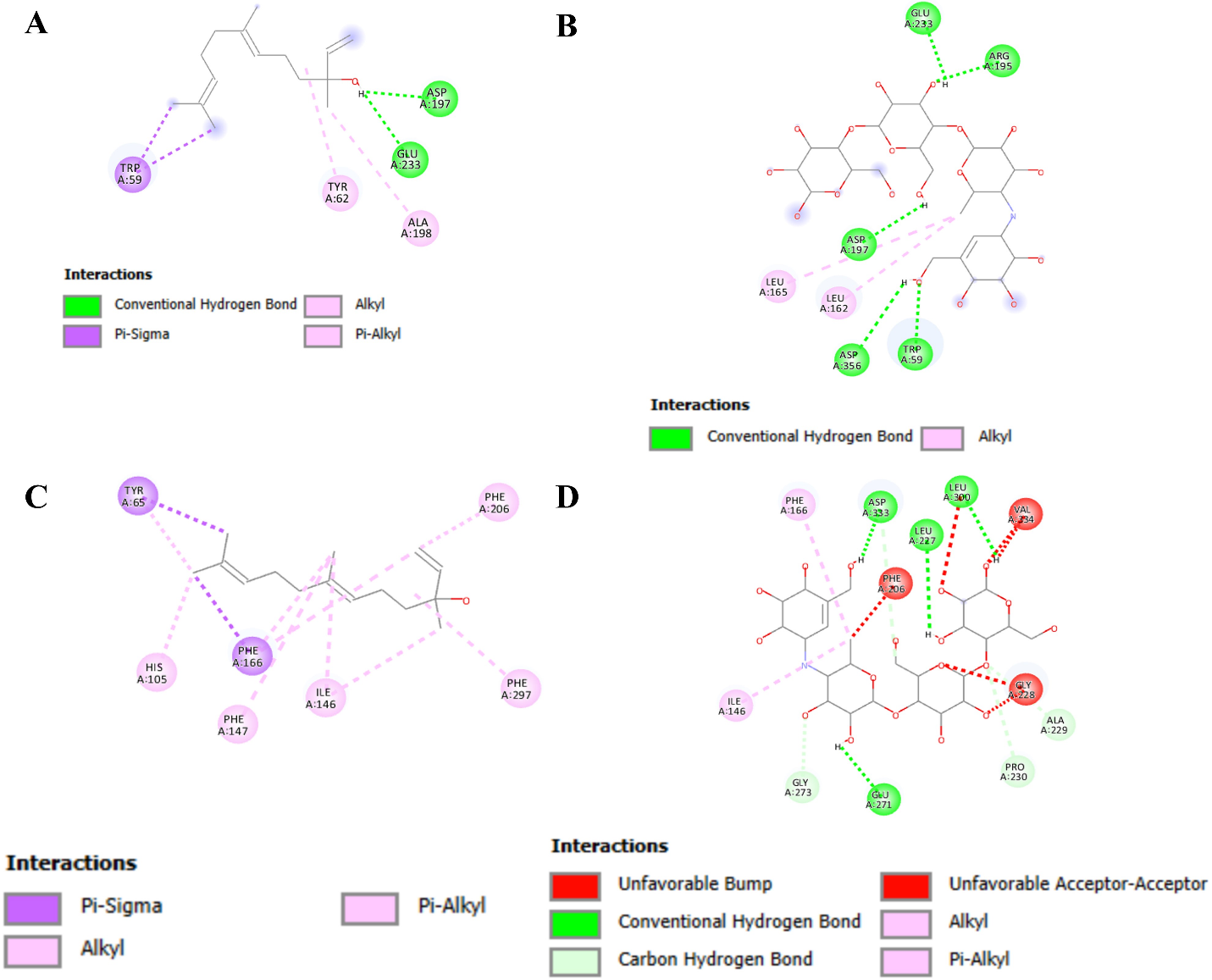


**Fig. SI 2:** 2-D interaction plot of the docked nerolidol into the active site of α-amylase A) nerolidol, B) acarbose, and α-glucosidase C) nerolidol, D) acarbose


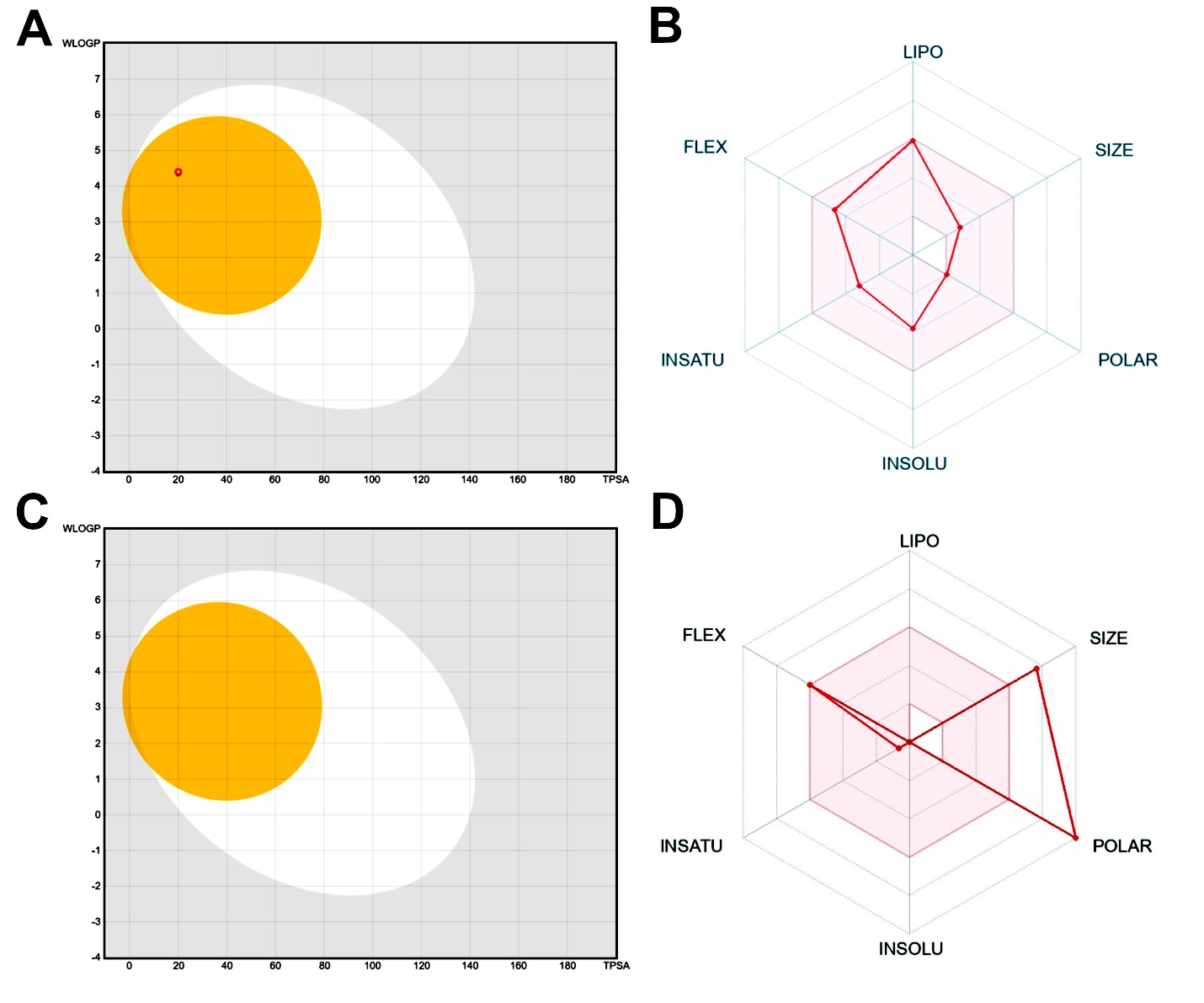


**Fig. SI 3:** A) Boiled-egg graphical representation of nerolidol, B) Bioavailability radar of nerolidol, C) Boiled-egg graphical representation of acarbose, D) Bioavailability radar of acarbose predicted using the swissADME tool
